# Radiomic Features of MRI Subcompartments Associate with Angiogenic and Inflammatory Transcriptomic Programs in Glioblastoma: An IvyGAP Exploratory Analysis

**DOI:** 10.3390/cancers18081293

**Published:** 2026-04-19

**Authors:** Daniele Piccolo, Marco Vindigni

**Affiliations:** Unit of Neurosurgery, Department of Head-Neck and Neuroscience, Azienda Sanitaria Universitaria Friuli Centrale, Presidio Ospedaliero Universitario Santa Maria della Misericordia, Piazzale Santa Maria della Misericordia, 15, 33100 Udine, Italy; marco.vindigni@asufc.sanita.fvg.it

**Keywords:** glioblastoma, radiomics, transcriptomics, tumor heterogeneity, inflammatory response

## Abstract

Background: Glioblastoma exhibits profound intratumoral heterogeneity, with anatomically distinct tumor zones characterized by divergent molecular programs that drive therapy resistance. Whether magnetic resonance imaging (MRI)-derived radiomic features can capture these regional transcriptomic differences remains unknown. We aimed to determine whether subcompartment-level radiomic features associate with transcriptomic pathway enrichment scores derived from biologically approximate tumor zones. Methods: We matched 28 patients (mean age 58.5 years; 13/28 *MGMT* methylated) across the IvyGAP RNA-seq atlas and the IVYGAP-RADIOMICS datasets. Single-sample GSEA (ssGSEA) pathway scores were computed for 24 gene sets. Radiomic features (3920 per subcompartment) were reduced to 597. Nested leave-one-patient-out cross-validation (LOPO-CV) with Elastic Net served as the primary predictive analysis; linear mixed-effects models (LMM) provided exploratory associational analysis. Analyses used a biologically motivated but spatially non-co-registered zone-to-subcompartment mapping; all reported associations are zone-approximate. Results: Twenty-one of 24 pathways showed no predictive signal (R^2^_cv_ ≤ 0). Inflammatory Response (R^2^_cv_ = 0.185, 95% CI [0.071, 0.355], *p* = 0.008) was the only pathway supported by both the nested CV (FDR = 0.096) and the exploratory LMM (FDR = 0.024, ΔR^2^ = 0.214 beyond subcompartment effects) analyses; the LMM association was robust to clinical covariate adjustment (likelihood ratio test *p* = 0.004). Angiogenesis (R^2^_cv_ = 0.209, 95% CI [0.028, 0.353], *p* = 0.006) reached nested CV significance (FDR = 0.096) but was not corroborated by the LMM (FDR = 0.445); it is therefore reported as a tentative single-framework signal requiring independent validation. T2-derived texture features were selected in 100% of folds for both pathways. Conclusions: Inflammatory Response is the only pathway supported by both analytical frameworks; Angiogenesis is a tentative nested-CV-only signal pending independent validation. The absence of signal for 21 of 24 pathways should not be interpreted as evidence of biological inaccessibility: at N = 28 (vs. N ≈ 240 required by Riley criteria), severe underpowering, attenuation from the non-spatial zone-to-subcompartment mapping, and methodological constraints each independently suffice to suppress real associations. Five of the 24 gene sets (the IvyGAP zone modules) are non-independent from the outcome data and cannot be interpreted as discovery. All reported associations are zone-approximate and may partly reflect macro-compartment (between-subcompartment) effects; validation in larger cohorts with spatially precise co-registration is essential.

## Simple Summary

Glioblastoma, the most aggressive brain cancer, consists of distinct biological regions that contribute to treatment resistance. Magnetic resonance imaging can differentiate tumor subregions based on their appearance, while gene expression profiling can uncover the molecular programs active in each area. Whether imaging-derived measurements accurately reflect these underlying molecular programs remains unknown. We combined two publicly available datasets for 28 glioblastoma patients, one providing imaging features from tumor subregions and another offering gene expression data from surgically isolated tissue zones. Of 24 molecular programs tested, inflammatory response was the only program supported by both analytical frameworks. Angiogenesis reached significance only in one framework and is reported as a tentative signal that requires independent validation. The remaining programs showed no detectable association; at this small sample size (28 patients vs. several hundred that would be needed for stable modeling), the absence of signal most likely reflects limited statistical power and the non-spatial nature of our zone-to-imaging mapping, rather than a true absence of biological association. Five of the 24 gene sets were derived from the same data used to test them and therefore do not constitute independent evidence. These findings delimit what can and cannot be inferred from imaging-based molecular profiling of brain tumors in small cohorts and motivate validation in larger, spatially co-registered datasets.

## 1. Introduction

Glioblastoma (GBM) is the most common and aggressive primary malignant brain tumor in adults, with a median overall survival of approximately 15 months despite maximal safe resection, temozolomide chemotherapy, and radiotherapy [1,2]. A defining feature of GBM is profound intratumoral spatial heterogeneity, with molecularly and histologically distinct regions coexisting within the same tumor [3,4]. This heterogeneity drives therapy resistance by harboring treatment-refractory cellular subpopulations and creating diverse microenvironmental niches [5]. Understanding the molecular programs that govern these distinct tumor regions is therefore critical for developing spatially informed therapeutic strategies.

The Ivy Glioblastoma Atlas Project (IvyGAP) represents a landmark effort to characterize this spatial heterogeneity at the transcriptomic level [6]. In IvyGAP, laser microdissection (LMD) was performed on tumor sections from 41 patients to isolate RNA from five histologically defined anatomic zones: cellular tumor (CT), microvascular proliferation (CTmvp), pseudopalisading cells adjacent to necrosis (CTpan), infiltrating tumor (IT), and leading edge (LE). The resulting 270 RNA-sequencing samples provide a spatially resolved transcriptomic atlas of GBM. Separately, the IVYGAP-RADIOMICS companion dataset [7] provides 3920 International Biomarker Standardization Initiative (IBSI)-compliant radiomic features per MRI-defined subcompartment (enhancing tumor [ET], non-enhancing tumor [NET], and peritumoral edema [ED]) for 31 of these patients with available multiparametric MRI (T1, T1-gadolinium, T2, FLAIR). Despite both datasets being publicly available since 2020, no study has linked the IVYGAP-RADIOMICS feature set to the zone-level IvyGAP RNA-seq data to test whether radiomic features reflect the transcriptomic programs of specific anatomic zones.

Prior studies have explored imaging-transcriptomic associations in GBM [8,9,10,11,12], including radiomic prediction of immune enrichment scores [13] and radiomic–genomic survival models [14,15], but none have connected zone-level transcriptomic data with IBSI radiomic features. Park et al. [8] used ADC/CBV clustering in only five patients (r < 0.30); Le et al. [9] and Zhang et al. [12] used whole-tumor approaches; Hu et al. [10] achieved spatially matched biopsies in a different dataset; Beig et al. [11] linked IvyGAP imaging subcompartments to bulk TCGA gene expression and ssGSEA for survival prediction, but did not use zone-level RNA-seq or transcriptomic pathway scores as outcomes. No study has linked the IvyGAP atlas with the IVYGAP-RADIOMICS feature set.

In this study, we test whether radiomic features extracted from MRI-defined tumor subcompartments (ET, NET, ED) associate with transcriptomic pathway enrichment scores derived from the biologically approximate IvyGAP anatomic zones. We employ a biologically motivated but spatially approximate zone-to-subcompartment mapping (e.g., CT and CTmvp to ET; CTpan to NET; IT and LE to ED) and use both linear mixed-effects models (associational analysis) and nested cross-validated Elastic Net regression (predictive analysis) as complementary analytical frameworks. We explicitly frame this as a hypothesis-generating exploratory analysis, acknowledging that the absence of voxel-level spatial co-registration between LMD sites and MRI subcompartments is a fundamental limitation that attenuates all observed associations.

## 2. Materials and Methods

### 2.1. Datasets and Patient Matching

Two publicly available datasets were used. The IvyGAP RNA-seq dataset was obtained from the Allen Institute for Brain Science portal and comprises 270 LMD samples from 41 patients across five anatomic zones (CT, CTmvp, CTpan, IT, LE) [6]. All IvyGAP patients are *IDH*-wildtype GBM (pre-WHO 2016 cohort). Gene expression values were provided as fragments per kilobase of transcript per million mapped reads (FPKM). The IVYGAP-RADIOMICS dataset was obtained from The Cancer Imaging Archive (TCIA) and provides 3920 IBSI-compliant radiomic features per subcompartment (ET, NET, ED) across four MRI sequences for 31 patients [7]. All MRI data originate from a single institution, eliminating the need for ComBat harmonization. Radiomic features were pre-extracted by Pati et al. [7] from BraTS-style tumor segmentations.

**Figure 1:**
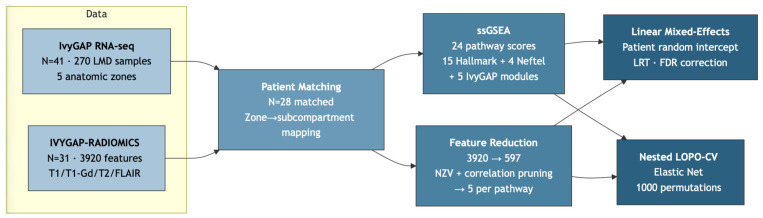
Study design. LMD = laser microdissected; ssGSEA = single-sample gene set enrichment analysis; NZV = near zero variance; LRT = likelihood ratio test; FDR = false discovery rate; LOPO-CV = leave-one-patient-out cross-validation.

Patient identifiers were matched across the two datasets, yielding 28 patients with both transcriptomic and radiomic data (mean age 58.5 years, SD 7.8; median Karnofsky Performance Status [KPS] 90; *MGMT* promoter methylated 13/28 [46%]; 27 primary tumors, 1 recurrent). Sex distribution is not reported in the public IvyGAP metadata. Zone availability varied across patients (CT available for all 28; other zones for 7–15), resulting in an unbalanced design after subcompartment aggregation (Section 3.1). Under WHO 2021 criteria, all patients classify as glioblastoma, *IDH*-wildtype (*IDH* status confirmed via the IvyGAP molecular annotations). Detailed prior treatment history (chemotherapy, radiation, dexamethasone) was not available from the IvyGAP metadata. Because CTmvp samples were available for only 9 of 28 patients, the ET transcriptomic score represents CT alone for 19 patients and mean (CT, CTmvp) for 9. This compositional inconsistency is particularly relevant for the Angiogenesis pathway, as CTmvp is the Angiogenesis-enriched zone; sensitivity analysis S1a (Section 2.9) addresses this by restricting ET to CT only. The overall study design is summarized in Figure 1.

### 2.2. Zone-to-Subcompartment Mapping

Because no spatial fiducials or validated registration pipeline links IvyGAP LMD sampling sites (identified post hoc on hematoxylin and eosin histology by neuropathologists [6]) to preoperative MRI voxels, we employed a biologically motivated but spatially approximate mapping between IvyGAP zones and MRI subcompartments (Table 1).

The biological rationale for each mapping is as follows. CT and CTmvp were mapped to ET because the cellular tumor core is the principal site of blood–brain barrier disruption and active Angiogenesis, producing gadolinium enhancement on T1-weighted imaging; CTmvp (microvascular proliferation) directly drives the leaky neovasculature responsible for contrast enhancement. CTpan was mapped to NET because pseudopalisading necrosis surrounds the devitalized necrotic core, corresponding to the non-enhancing central region on MRI where tissue is no longer viable enough to support contrast uptake. IT and LE were mapped to ED because infiltrating tumor cells and the leading edge of invasion extend beyond the enhancing margin into the peritumoral region, which appears hyperintense on T2/FLAIR due to vasogenic edema from tumor-induced blood–brain barrier disruption.

Park et al. [8] reported weak but directionally consistent correlations between histologic zone proportions and MRI subcompartment volumes (mean r = 0.242), implying substantial signal attenuation (r^2^ = 0.059). All reported associations must be interpreted as zone-approximate, not spatially precise, and the degree of attenuation cannot be precisely estimated.

### 2.3. Transcriptomic Target Definition

Twenty-four gene sets were used to compute single-sample Gene Set Enrichment Analysis (ssGSEA) pathway enrichment scores for each LMD sample [16,17]: (1) fifteen GBM-relevant Hallmark gene sets from the Molecular Signatures Database (MSigDB): Hypoxia, Angiogenesis, Epithelial–Mesenchymal Transition (EMT), Inflammatory Response, TNF-alpha/NF-kB Signaling, IL-6/JAK/STAT3 Signaling, Interferon Gamma Response, *P53* Pathway, *MYC* Targets V1, *E2F* Targets, G2M Checkpoint, mTORC1 Signaling, Glycolysis, Oxidative Phosphorylation, and Complement; (2) four Neftel et al. [5] cellular state signatures: mesenchymal-like (MES; MES1 and MES2 collapsed), astrocyte-like (AC), oligodendrocyte progenitor-like (OPC), and neural progenitor-like (NPC; NPC1 and NPC2 collapsed, as sub-states share core transcription factor programs and the sample size limits power to distinguish sub-state nuances); and (3) five IvyGAP zone-specific gene modules defined as the top 200 differentially expressed genes per zone (Wilcoxon test, FDR < 0.05, |log_2_ fold change| > 1).

ssGSEA was computed on log_2_(FPKM + 1)-transformed expression values using the GSVA package [17] with Gaussian kernel cumulative density function estimation. Zone-level enrichment scores were aggregated to the subcompartment level by computing the mean across zones mapped to each subcompartment (Table 1).

Five of the 24 gene sets used in this analysis—the IvyGAP CT, CTmvp, CTpan, IT, and LE zone modules—were derived directly from the same IvyGAP transcriptomic atlas that provides our outcome data. This introduces a structural non-independence between gene-set definition and outcome evaluation: associations involving these five modules cannot be interpreted as independent biological discovery and are reported as internal-consistency checks only. The 15 Hallmark and four Neftel signatures—wholly external to IvyGAP—therefore constitute the interpretively primary gene sets for all downstream inference. This circularity is revisited as a dedicated subsection in the main Discussion (Section 4.5). Pairwise Jaccard similarity indices were computed for all 24 gene sets to quantify redundancy (Section 3.9).

### 2.4. Feature Reduction Pipeline

The 3920 radiomic features per subcompartment were reduced through a two-stage unsupervised filtering pipeline: (1) near-zero-variance filtering using the nearZeroVar function from the caret package [18], removing features with near-zero variance ratios (3920 to 3860 features); and (2) pairwise Spearman correlation filtering, removing one feature from each pair with |r| > 0.90 (3860 to 597 features). These two steps are unsupervised (outcome-agnostic) and do not introduce data leakage.

For the associational analysis (Section 2.5), a third supervised step was applied: per-pathway univariate Spearman correlation with each pathway enrichment score within each subcompartment separately, retaining features with minimum FDR < 0.10 across subcompartments (Benjamini–Hochberg correction [19] applied within each subcompartment) and selecting the top five per pathway. This step was performed on the full dataset, which is standard practice for exploratory associational analyses but introduces potential optimistic bias that must be acknowledged. After this pipeline, 12 of 24 pathways had at least one radiomic feature and were carried forward to mixed-effects modeling. For the predictive analysis (Section 2.6), supervised feature selection was instead performed inside each cross-validation fold to prevent data leakage. The complete feature reduction pipeline is illustrated as a flowchart in Figure S10, with dimensionality at each stage shown in Figure S9.

### 2.5. Exploratory Associational Analysis: Linear Mixed-Effects Models

The LMM analysis is exploratory and employs full-dataset feature pre-selection (Section 2.4), which may yield optimistically biased results. The nested CV (Section 2.6) serves as the primary evidentiary framework because feature selection occurs inside each fold, eliminating data leakage.

For each of the 12 pathways with at least one radiomic feature, a linear mixed-effects model (LMM) was fitted using the lme4 package [20] with lmerTest [21] for denominator degrees of freedom (Satterthwaite approximation):

Full model: z_pathway ~ z_rad_1 + … + z_rad_k + subcompartment + (1 | patient_id)

Null model: z_pathway ~ subcompartment + (1 | patient_id)

where z_pathway denotes the globally z-scored pathway enrichment score, z_rad_1 through z_rad_k denote the within-subcompartment z-scored radiomic features (k ≤ 5), subcompartment is a fixed factor (ET, NET, ED), and patient_id is a random intercept to account for repeated measures within patients.

The omnibus radiomic contribution was assessed via likelihood ratio test (LRT) comparing full and null models. Marginal R^2^ and conditional R^2^ were computed using the Nakagawa–Schielzeth method [22,23]. FDR correction was applied across all 24 pathways [19], assigning *p* = 1.0 to 12 pathways with zero features. This conservative approach inflates the FDR denominator, making it harder to achieve significant results, but does not correspond to a formal BH-FDR guarantee, since the assigned values are not true *p*-values under the null. For FDR-significant pathways, coefficient *p*-values were Holm-corrected [24]. Two FDR thresholds are used, reflecting the different statistical contexts. For the LMM, where only 12 pathways with surviving features were tested, conventional FDR < 0.05 was applied. For nested CV, the conservative 24-pathway denominator (assigning *p* = 1.0 to all pathways with R^2^_cv_ ≤ 0) yields stringent FDR values; FDR < 0.10 was therefore considered the interpretive threshold for this exploratory analysis. Feature pre-selection may inflate LMM significance; this is independently evaluated through nested CV (Section 2.6). For transparency, we also report the standard Benjamini–Hochberg FDR correction restricted to the pathways actually tested (12 for LMM; 3 with R^2^_cv_ > 0 for nested CV) as a comparison, in addition to the conservative 24-pathway correction.

### 2.6. Primary Predictive Analysis: Nested Cross-Validated Elastic Net

Elastic Net regression [25] was selected because it combines L1 and L2 penalties, promoting grouped selection of correlated features while maintaining sparsity [26], addressing the instability of LASSO [27] in settings with highly correlated radiomic features. The conservative lambda.1se rule was used to guard against overfitting [28].

To provide an unbiased assessment free from data leakage, we implemented nested leave-one-patient-out cross-validation (LOPO-CV) for all 24 pathways. Radiomic features and pathway scores were averaged across subcompartments within each patient, yielding N = 28 independent observations. Patient-level averaging sacrifices subcompartment-level resolution; the LMM (Section 2.5) directly models this variation and is complementary.

For each LOPO fold (28 folds per pathway): (1) one patient was held out for testing; (2) univariate Spearman correlations were computed between each of the 597 candidate features and the pathway score using only the 27 training patients; (3) features with FDR < 0.10 were retained, and the top five by raw *p*-value were selected; and (4) Elastic Net regression [25] was fitted on the selected features with alpha grid search (0.1 to 1.0, step 0.1) and lambda selected at the conservative one-standard-error rule (lambda.1se), using inner 5-fold CV within the training set.

Performance was evaluated via R^2^_cv_, MAE, and Spearman correlation. Uncertainty was quantified via nonparametric bootstrap confidence intervals; the nested permutation *p*-values (Section 2.7) are the primary inferential tool. Implementation details of the bootstrap procedure and its interpretive limits are provided in Supplementary Methods S1.

Feature stability was assessed by recording which features were selected in each LOPO fold. Features appearing in more than 50% of folds were designated as “stability-selected” [29]. Hyperparameter selection details and stability of alpha/lambda across folds are reported in Supplementary Methods S1 and Supplementary Table S11.

### 2.7. Permutation Testing

Two permutation frameworks were employed. For the LMM associational analysis, patient-level permutation (N = 1000) shuffled the mapping between patients’ pathway scores and radiomic features, preserving the within-patient correlation structure. The LRT chi-squared statistic served as the primary test statistic.

For the nested CV predictive analysis, a fully nested permutation test (N = 1000) was performed for pathways with R^2^_cv_ > 0. In each permutation, pathway scores were shuffled across patients, and the entire nested CV pipeline, including feature selection inside each fold, was re-executed. This provides a truly unbiased permutation *p*-value that accounts for the adaptive feature selection process. Permutation *p*-values were computed as n_extreme_/N_perm_.

### 2.8. Legacy Analysis

A legacy analysis with pre-screened features (selected on the full dataset before cross-validation) is reported in Supplementary Table S5 for methodological comparison with the bias-free nested CV.

### 2.9. Sensitivity Analyses

Ten pre-specified sensitivity analyses tested robustness to alternative zone mappings (S1a, S1b), patient exclusion (S2), aggregation method (S3), FDR subset correction (S4a–c), random effects structure (S6), standardization approach (S7), denominator degrees of freedom (S8), and clinical covariate adjustment for Inflammatory Response (age, *MGMT* status, Verhaak molecular subtype; S9). Full details are in Supplementary Table S3.

### 2.10. Sample Size Justification

The minimum sample size for *p* = 5 predictors, R^2^ = 0.20, and shrinkage S ≥ 0.90 was N_min_ = 240 (pmsampsize [30], Riley et al. [31], Criterion 4). With N = 28, the minimum detectable R^2^ at S ≥ 0.90 was 0.641.

### 2.11. Software and Reproducibility

All analyses were conducted in R version 4.5.0 using the following key packages: lme4 v1.1-37 [20], lmerTest v3.1-3 [21], performance v0.13.0 [23], glmnet v4.1-8 [25], pmsampsize v1.1.3 [30], GSVA [17], caret v7.0-1 [18], and pbkrtest v0.5.5 (for Kenward-Roger correction). A random seed of 42 was used for all stochastic procedures. A CLEAR (CheckList for EvaluAtion of Radiomics research) [32] compliance table is provided as Supplementary Table S8.

## 3. Results

Throughout, R^2^_cv_ denotes cross-validated R^2^, R^2^_m_ marginal R^2^ (fixed effects), R^2^_c_ conditional R^2^ (fixed + random), and ΔR^2^_m_ the radiomic increment beyond subcompartment effects.

### 3.1. Data Availability and Feature Reduction

Of 41 IvyGAP patients and 31 IVYGAP-RADIOMICS patients, 28 were present in both datasets and constituted the analysis cohort. Of these, 27 (96%) underwent primary surgery and one (4%) had recurrent tumor (mean age 58.5 years, SD 7.8; median KPS 90; *MGMT* methylated 13/28). These 28 patients contributed a total of 50 observations across three MRI subcompartments (ET: n = 28; NET: n = 15; ED: n = 7), with only 6 of 28 patients having data for all three subcompartments. The imbalance reflects differential availability of zone-level RNA-seq data across patients (Figure S8).

The unsupervised radiomic feature reduction pipeline progressively reduced dimensionality: 3920 initial features were filtered to 3860 after near-zero-variance removal, and to 597 after Spearman correlation pruning (|r| > 0.90) (Figure S9).

### 3.2. Nested Cross-Validation: Predictive Performance

**Figure 2:**
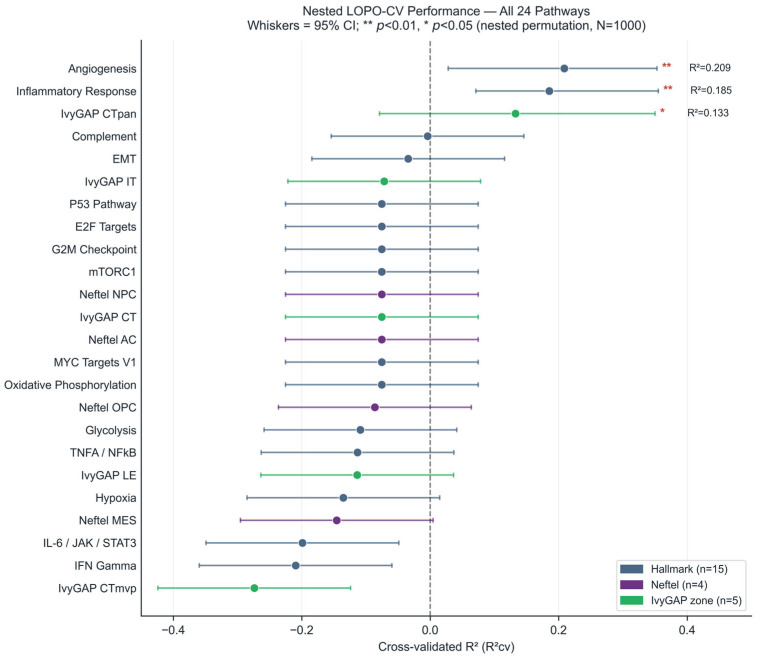
Nested cross-validated predictive performance of radiomic features for 24 transcriptomic pathway enrichment scores. Each point shows R^2^_cv_ from leave-one-patient-out cross-validation (LOPO-CV; N = 28 patients) with Elastic Net regression and feature selection performed independently inside each fold. Whiskers indicate bootstrap 95% confidence intervals. Points are colored by gene set category: dark blue = Hallmark pathways (n = 15), purple = Neftel cellular states (n = 4), green = IvyGAP zone modules (n = 5). Significance annotations denote nested permutation *p*-values (** *p* < 0.01, * *p* < 0.05; N = 1000). Pathways are ordered by descending R^2^_cv_. The dashed vertical line marks R^2^_cv_ = 0. R^2^_cv_ = cross-validated coefficient of determination.

Of the 24 pathways evaluated, three showed positive predictive signal (R^2^_cv_ > 0) in the nested LOPO-CV analysis with internal feature selection (Table 2, Figure 2).

After BH-FDR correction across all 24 pathways, Angiogenesis and Inflammatory Response both reached FDR < 0.10 (FDR = 0.096), while the CTpan module did not (FDR = 0.104). Under a standard BH-FDR correction restricted to the three pathways with R^2^_cv_ > 0, both Angiogenesis and Inflammatory Response reached FDR = 0.012; CTpan reached FDR = 0.013 but should still be interpreted cautiously due to its circularity and composition effect. Based on convergent evidence from the complementary LMM analysis (Section 3.4), which identified Inflammatory Response as the sole FDR-significant pathway (LMM FDR = 0.024) while Angiogenesis did not reach LMM significance (FDR = 0.445), Inflammatory Response was designated as the primary pathway. The Angiogenesis finding is retained and reported as a tentative nested-CV-only signal: because it is supported by only one of the two analytical frameworks and feature selection is known to be unstable at N = 28, it requires independent validation before any biological conclusion can be drawn.

The CTpan module had a confidence interval spanning zero, is circular by construction (Section 2.3, Limitation 7), and showed a significant composition effect (Spearman rho = 0.579, *p* = 0.001); it should not be considered robust. Composition baseline tests for Angiogenesis (rho = −0.105, *p* = 0.594) and Inflammatory Response (rho = −0.003, *p* = 0.988) were non-significant.

The remaining 21 of 24 pathways showed no predictive signal (R^2^_cv_ ≤ 0; Table S1). These included all four Neftel cellular state signatures (MES, AC, OPC, NPC), four of five IvyGAP zone-specific modules (CT, CTmvp, IT, LE), and 13 Hallmark pathways (Hypoxia, EMT, TNF-alpha/NF-kB Signaling, IL-6/JAK/STAT3 Signaling, Interferon Gamma Response, *P53* Pathway, *MYC* Targets V1, *E2F* Targets, G2M Checkpoint, mTORC1 Signaling, Glycolysis, Oxidative Phosphorylation, and Complement). Of these, 12 pathways had zero features passing the univariate filter in any fold (median features per fold = 0). This pattern is consistent with an absence of detectable patient-level association under the present design but does not demonstrate biological inaccessibility: at N = 28 (vs. N ≈ 240 required), feature-selection instability and attenuation from the non-spatial zone-to-subcompartment mapping (Park et al. [8] r^2^ ≈ 0.059) are each sufficient to suppress real associations of moderate effect size. The dominant negative finding and its alternative explanations are discussed in Section 4.

### 3.3. Feature Stability and Identification

**Figure 3:**
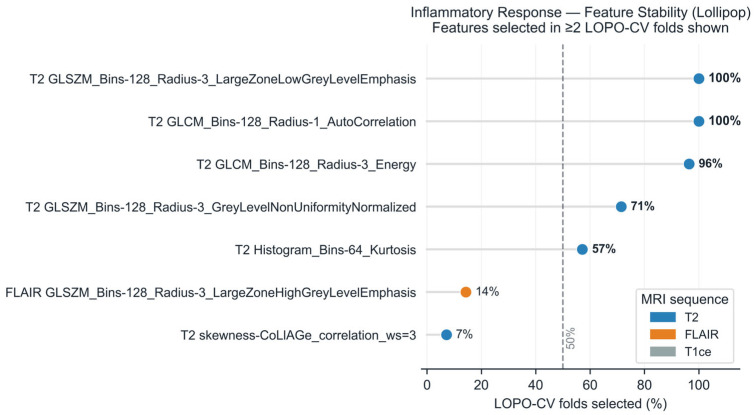
Feature stability for the Inflammatory Response pathway across 28 LOPO-CV folds. Each row represents a radiomic feature selected in at least two folds; the *x*-axis shows selection frequency (percentage of folds in which the feature was selected by univariate Spearman screening, FDR < 0.10, top 5). Points are colored by MRI sequence: blue = T2, orange = FLAIR, gray = T1ce. The dashed vertical line marks the 50% stability threshold. LOPO-CV = leave-one-patient-out cross-validation; GLCM = Gray-Level Co-occurrence Matrix; GLSZM = Gray-Level Size Zone Matrix.

All features contributing to the Inflammatory Response prediction were T2-derived (Table S12, Figure 3), consistent with the known sensitivity of T2-weighted imaging to inflammatory edema and tissue water content.

Two features were selected in all 28 folds (100% stability): T2 GLCM AutoCorrelation (rad_1707) and T2 GLSZM Large Zone Low Grey Level Emphasis (Bins-128, Radius-3; rad_1950). A third GLCM feature (Energy, rad_1930) was selected in 96% of folds (Table S12, Figure 3). Note that rad_1950 (Bins-128, Radius-3) and the LMM feature rad_1732 (Bins-128, Radius-1) are distinct radius configurations of the same GLSZM texture class, explaining their independent selection across the two analytical frameworks.

In imaging terms, GLCM AutoCorrelation quantifies the consistency of local T2 signal patterns—high values indicate homogeneous tissue, while low values reflect heterogeneous signal mixtures. GLSZM Large Zone Low Grey Level Emphasis captures the extent of large contiguous regions with low T2 signal intensity; its negative association with inflammatory enrichment (beta = −0.471 in the LMM; Section 3.4) suggests that greater inflammatory activity corresponds to more heterogeneous tissue architecture with fewer large homogeneous zones. GLCM Energy measures the uniformity of the T2 signal distribution. Together, these features quantify the heterogeneity of T2 signal within tumor subcompartments, consistent with the patchy edema, macrophage infiltration, and vascular permeability changes characteristic of the inflammatory microenvironment.

For the Angiogenesis pathway, stability selection identified five features in more than 50% of LOPO folds (Table S9), all T2-derived: three GLSZM texture features (100% stability) and two first-order/GLSZM features (96% stability).

A complete feature lookup table mapping all radiomic feature indices to their full IBSI names is provided in Supplementary Table S6.

### 3.4. Associational Analysis: Mixed-Effects Models

Of the 12 pathways tested in the LMM analysis (after supervised univariate pre-screening), only Inflammatory Response reached significance after FDR correction across all 24 pathways: FDR = 0.024, marginal R^2^ = 0.384, of which ΔR^2^ = 0.214 is attributable to radiomic features beyond the subcompartment effect (null model R^2^_m_ = 0.170), conditional R^2^ = 0.687, LRT χ ^2^ = 20.53 (df = 5, *p* = 0.001) (Table 3, Figure S1). Note that features were pre-selected on the full dataset; marginal R^2^ values are likely optimistically biased (see Section 2.4). The intraclass correlation for Inflammatory Response was ICC = 0.492, indicating moderate within-patient correlation.

Pathways with high R^2^_m_ (full) but minimal ΔR^2^_m_, such as Hypoxia (ΔR^2^_m_ = 0.010) and Glycolysis (ΔR^2^_m_ = 0.015), showed negligible radiomic increments. Inflammatory Response had the largest ΔR^2^_m_ (0.214).

Residual diagnostics (Figure S3) showed approximate normality (Shapiro–Wilk *p* = 0.384). One observation exceeded Cook’s D = 1.0 (D = 1.097); sensitivity analyses (S1a, S2) confirm robust results (Table S3).

Angiogenesis had only one feature surviving the univariate pre-screen and did not reach LMM FDR significance (FDR = 0.445), but the nested CV analysis identified a more informative feature set, underscoring the importance of the nested CV approach.

The Inflammatory Response model included five radiomic features (Table S13, Figure S2). The strongest individual contributor was T2 GLSZM Large Zone Low Grey Level Emphasis (Bins-128, Radius-1; rad_1732; beta = −0.471, 95% CI [−0.758, −0.184], Holm-adjusted *p* = 0.010), indicating a negative association between this texture feature and inflammatory pathway enrichment (Table S13). One additional feature, T2 CoLIAGe skewness of difference variance (ws = 5; rad_1080), showed nominal significance (beta = 0.308, 95% CI [0.007, 0.610], uncorrected *p* = 0.045) but did not survive Holm correction (adjusted *p* = 0.181).

The subcompartment fixed effect was also significant (F(2, 17.5) = 9.48, *p* = 0.002), indicating that inflammatory pathway enrichment scores differed across ET, NET, and ED subcompartments independently of radiomic features.

### 3.5. Permutation Testing

Patient-level permutation testing for the LMM (N = 1000 permutations) assessed whether the Inflammatory Response LRT χ^2^ statistic exceeded chance expectation. The observed χ^2^ of 20.53 yielded a permutation *p*-value of 0.055 for the χ^2^ statistic and *p* = 0.050 for marginal R^2^ (Figure S4). These values indicate that the Inflammatory Response association exceeds approximately 95% of the null distribution but does not reach conventional significance at α = 0.05. This permutation test used pre-selected features (i.e., the same features in every permutation), which does not fully account for the adaptive feature selection process.

The nested permutation test (Section 2.7), which re-selects features inside each fold of each permutation, provides a more rigorous assessment. All three pathways with R^2^_cv_ > 0 reached significance at the uncorrected level: Angiogenesis (*p* = 0.006), Inflammatory Response (*p* = 0.008), and IvyGAP CTpan module (*p* = 0.013) (Table 2). After BH-FDR correction across all 24 pathways (Table S10), Angiogenesis and Inflammatory Response reached FDR < 0.10 (FDR = 0.096 each), while CTpan did not (FDR = 0.104). The decision to test only pathways with R^2^_cv_ > 0 in the nested permutation is data-dependent; this is accounted for by reporting FDR-corrected *p*-values across all 24 pathways.

### 3.6. Clinical Covariate Adjustment

To assess whether the radiomic–transcriptomic association for Inflammatory Response was confounded by clinical variables, we progressively added covariates to the LMM (Table S7).

The radiomic–transcriptomic association for Inflammatory Response remained significant across all covariate models (LRT *p* < 0.005 in all cases). Adding age and *MGMT* methylation had minimal impact on the marginal R^2^ (Models B-C), while adding Verhaak molecular subtype (Model D) increased the marginal R^2^ to 0.479, suggesting that molecular subtype explains additional variance in inflammatory pathway activity beyond radiomic features alone. Critically, the radiomic contribution remained significant even in the fully adjusted model (LRT *p* = 0.004), indicating that the association is not driven by confounding from age, *MGMT* status, or molecular subtype. Model D results should be interpreted with caution, given the high parameter-to-observation ratio (12 parameters from 50 observations across 28 clusters).

### 3.7. Sensitivity Analyses

The Inflammatory Response association was robust across the majority of sensitivity analyses (Table S3, Figure S5). The LRT *p*-value remained below 0.01 under alternative zone mappings (S1a: *p* = 0.003; S1b: *p* = 0.003), exclusion of patients with single subcompartments (S2: *p* = 0.007), median aggregation (S3: *p* = 0.001), Hallmark-only FDR re-correction (S4a: FDR = 0.010), and Kenward–Roger denominator degrees of freedom (S8: *p* = 0.006).

Robustness under S1a (ET = CT only, excluding CTmvp) addresses the concern that CTmvp samples could confound the ET signal (S1a: *p* = 0.003, R^2^_m_ = 0.365).

Two analyses qualified the primary result. Global standardization (S7) weakened the association to *p* = 0.076, indicating that the radiomic signal captures within-subcompartment variation in tissue properties rather than between-subcompartment differences, which are already captured by the subcompartment fixed effect. The random slopes model (S6) could not be fitted due to insufficient sample size.

### 3.8. Legacy Pre-Screened Analysis

The legacy pre-screened Elastic Net yielded R^2^_cv_ = −0.104 for Inflammatory Response (Table S5), compared with R^2^_cv_ = 0.185 from nested CV, confirming that pre-screening introduced overfitting.

### 3.9. Gene Set Overlap

Jaccard similarity analysis confirmed that Angiogenesis and Inflammatory Response represent independent signals (maximum J < 0.10). Only the IvyGAP IT and LE modules showed high overlap (J = 0.653).

## 4. Discussion

This exploratory analysis tested whether MRI-derived radiomic features from tumor subcompartments associate with regional transcriptomic programs in GBM, leveraging the IvyGAP and IVYGAP-RADIOMICS public datasets. Using a nested cross-validation framework, not employed in prior IvyGAP radiomic studies [8,9,10,11,12], we identified Inflammatory Response (R^2^_cv_ = 0.185) as the only transcriptomic program supported by both the nested CV (FDR = 0.096) and the complementary LMM (FDR = 0.024) analyses. Angiogenesis (R^2^_cv_ = 0.209) reached nested-CV significance (FDR = 0.096) but was not corroborated by the LMM (FDR = 0.445) and is therefore reported as a tentative signal requiring independent validation. Twenty-one of 24 pathways showed no signal. In the complementary exploratory associational analysis, Inflammatory Response was the sole pathway reaching FDR significance (FDR = 0.024 across all 24 pathways, ΔR^2^ = 0.214 beyond subcompartment effects), driven primarily by T2-derived texture features. Given the sample size of 28 patients, the zone-approximate mapping, and the absence of external validation, these associations should be regarded as preliminary hypotheses requiring prospective confirmation before any clinical translation. Furthermore, in the absence of voxel-level spatial co-registration between LMD sites and MRI voxels, the observed associations may partly reflect macro-compartment effects (systematic differences between ET, NET, and ED subcompartments) rather than true within-zone radiogenomic coupling; the R^2^ decomposition (Table 3) helps quantify the subcompartment contribution, but it cannot fully separate macro-compartment from true zone-level signal.

### 4.1. Biological Interpretation

The identification of Angiogenesis and Inflammatory Response as the two pathways with predictive radiomic signals is biologically plausible. Both processes directly modulate MRI signals through mechanisms that alter tissue contrast.

Angiogenesis drives gadolinium enhancement through leaky neovasculature. However, the dominant features were T2-derived texture features. This likely reflects a methodological constraint: within the ET subcompartment (defined by enhancement), T1-gadolinium features have a restricted dynamic range because the region is already selected for high enhancement, whereas T2 features retain full dynamic range and capture internal heterogeneity of the enhancing core. Biologically, T2 texture within the enhancing tumor reflects downstream consequences of Angiogenesis (irregular vascular architecture, patchy edema, and microhemorrhage) rather than the vascular process directly. This interpretation is consistent with Dextraze et al. [14], who identified associations between Angiogenesis-related pathways and MRI-defined imaging habitats in 85 GBM patients. However, we emphasize that the Angiogenesis finding in our cohort is supported by only one of the two analytical frameworks (nested CV R^2^_cv_ = 0.209; LMM FDR = 0.445); at N = 28, cross-validated R^2^ estimates and feature selection are both unstable, and asymmetric convergence between frameworks limits the robustness of any single-model result. The Angiogenesis signal should therefore be treated as hypothesis-generating rather than established, and any biological interpretation deferred to independent validation in a spatially co-registered cohort of adequate size.

Inflammatory Response involves tumor-associated macrophages, which constitute 30–50% of the GBM mass [33,34], modulating vascular permeability and blood–brain barrier integrity, processes that directly affect T2/FLAIR signal. The dominant feature (T2 GLSZM Large Zone Low Grey Level Emphasis, beta = −0.471) showed a negative association, suggesting that inflammatory activity corresponds to more heterogeneous tissue architecture.

### 4.2. Why These Pathways Survive Spatial Mismatch

The selective survival of Inflammatory Response is consistent with the spatial scale of this process. Tumor-associated macrophages constitute 30–50% of GBM mass [33,34], permeating the entire subcompartment and creating a spatially homogeneous alteration of tissue properties. Because inflammation modulates the full subcompartment volume, any LMD sample captures the inflammatory signal, and radiomic features integrate the same signal across the volume. The effective signal attenuation from spatial mismatch may therefore be lower than the 94% predicted by Park et al. [8] correlations for spatially localized processes.

Angiogenesis operates differently: neovascularization creates focal structures (glomeruloid microvascular proliferation bodies), but its downstream tissue-level consequences, such as edema, vascular permeability, and microhemorrhage, are diffuse and captured by T2 texture across the enhancing core. The mechanism is structural-consequential rather than diffuse per se.

### 4.3. Scope and Negative Results

The dominant finding is negative: 21 of 24 pathways showed no predictive signal (R^2^_cv_ ≤ 0). Before discussing patterns within this null, we state explicitly that the absence of signal should not be interpreted as evidence that these pathways are biologically inaccessible to MRI. Three alternative explanations—any of which is sufficient on its own to suppress real associations of moderate effect size—must be considered first. First, severe statistical underpowering: Riley criteria [31] for the intended effect size require N ≈ 240, whereas the present analysis uses N = 28; at this sample size feature selection and cross-validated R^2^ are both unstable. Second, attenuation introduced by the non-spatial zone-to-subcompartment mapping: Park et al.’s [8] empirical correlations (mean r = 0.242, r^2^ ≈ 0.059) imply roughly 94% loss of signal variance, so only very large true effects can survive. Third, methodological constraints: The restricted expressive capacity of hand-crafted IBSI features, the absence of voxel-level co-registration, and the structural non-independence of the five IvyGAP-derived zone modules (Section 4.5) all bias null findings toward spurious absence. A true biological null is possible but cannot be distinguished from these alternative explanations with the present data. The patterns discussed below are therefore presented as informative regularities, not as evidence of biological boundaries.

Four of five IvyGAP zone-specific gene modules showed zero features passing the univariate filter. Because these modules are structurally non-independent from the outcome data (Section 4.5), the absence of signal cannot be interpreted as a substantive biological finding about zone-module detectability and is most safely read as consistent with attenuation from the zone-to-subcompartment mapping; at N = 28, a genuine biological signal and a mapping-attenuation artifact cannot be empirically distinguished from the present data.

All four Neftel cellular state signatures (MES, AC, OPC, NPC) showed no signal in this cohort. These signatures represent cell-intrinsic transcription factor programs (e.g., *CEBP/D* for MES, *OLIG1/2* for OPC) that are not expected to directly alter tissue-level MRI contrast at scales resolvable by conventional radiomics in a sample of this size, unlike Angiogenesis or inflammation, which physically modulate vascular permeability and tissue water content. Furthermore, single-cell data show that all four states coexist within the same histological zone, and bulk RNA-seq from that zone averages across these states, which may reduce spatial specificity. The spatial scale gap is also substantial: Neftel states vary at the 10–50 micron single-cell level, two to three orders of magnitude below the millimeter-scale radiomic features. The selective failure of cell-intrinsic signatures in our data is compatible with the interpretation that radiomics preferentially captures tissue-level biology in this cohort, although we emphasize that the absence of signal should not be interpreted as a definitive absence of biological association. The lack of signal for these pathways may equally reflect the limited statistical power (N = 28), the attenuation introduced by the zone-approximate mapping, or the restricted expressive capacity of hand-crafted IBSI features, rather than a true absence of cell-level radiogenomic coupling. Distinguishing these explanations would require spatially co-registered data, substantially larger cohorts, and deep-learning-derived features.

Several Hallmark pathways with a biological rationale for MRI detectability, including Hypoxia, EMT, and Glycolysis, also showed no radiomic association. For Hypoxia, the high null-model R^2^_m_ (0.837) indicates that subcompartment membership alone captures the Hypoxia gradient, leaving only ΔR^2^_m_ = 0.010 for radiomic features; once the categorical subcompartment label is known, within-subcompartment radiomic variation adds virtually no information about Hypoxia status.

Three mechanistic factors likely explain why most pathways showed no signal. First, spatial scale mismatch: cell-intrinsic programs (Neftel states) vary at the 10–50 micrometer single-cell level, three to four orders of magnitude below the centimeter-scale volumes over which radiomic features are computed. Second, insufficient tissue-level contrast modulation: transcriptomic programs that do not alter tissue water content, vascularity, or cellularity—the physical properties governing MRI signal—are invisible to radiomics regardless of how strongly they are expressed. Third, the zone-to-subcompartment mapping attenuation (Park et al. [8] mean r = 0.242, r^2^ = 0.059) implies that approximately 94% of signal variance is lost in the mapping; only associations with very large true effect sizes could survive this attenuation at N = 28. Together, these factors explain why only tissue-scale microenvironmental processes (inflammation, Angiogenesis) that directly and diffusely modulate MRI contrast produced detectable signal, while the majority of pathways did not.

### 4.4. Methodological Considerations

Data leakage through feature pre-selection is a critical concern in radiomic studies [35]. Our legacy Elastic Net with pre-screened features produced R^2^_cv_ = −0.104 for Inflammatory Response, while the bias-free nested CV yielded R^2^_cv_ = 0.185, demonstrating that pre-selection introduced optimistic bias that paradoxically worsened predictions. We addressed this by implementing nested CV as the primary analysis, with feature selection inside each LOPO fold. For the LMM, features were pre-selected on the full dataset; the independent confirmation in nested CV provides convergent evidence that the Inflammatory Response association is not an artifact of data leakage. The discrepancy between LMM permutation *p* = 0.055 and nested CV permutation *p* = 0.008 for Inflammatory Response reflects different null hypotheses: the LMM permutation uses pre-selected features in every permutation, while the nested CV permutation re-executes the full pipeline, including feature selection under the null, generating a tighter null distribution. Importantly, the results are partly model-dependent. Angiogenesis reached FDR significance only in the nested CV analysis (FDR = 0.096 conservative; FDR = 0.012 standard BH on tested pathways) but not in the LMM (FDR = 0.445), while Inflammatory Response was significant in both frameworks (LMM FDR = 0.024). This asymmetric convergence—one pathway supported by both models, the other by only one—limits the robustness of the Angiogenesis finding and underscores that the results are not fully model-agnostic. Readers should interpret Angiogenesis as a nested-CV-only signal requiring independent replication before any biological conclusion can be drawn.

The designation of Inflammatory Response as the primary pathway was based on consistent results across the nested CV (FDR = 0.096) and LMM (FDR = 0.024) analyses. Because both analyses use the same 28 patients and the same ssGSEA outcome scores, the convergence of nested CV and LMM results represents consistency across different statistical frameworks applied to the same observations, not independent replication. Independent validation would require a separate patient cohort with spatially co-registered imaging and transcriptomics, which does not currently exist. The two approaches—one predictive with internal feature selection, one associational with pre-screened features—converge on the same pathway using different model structures and units of analysis (patient-level vs. observation-level), but this convergence increases internal consistency without substituting for external replication. The dependence of results on the standardization approach (S7: *p* = 0.076 under global standardization) suggests that the association is partly driven by within-subcompartment relative feature values rather than absolute magnitudes.

Five of the 24 gene sets we evaluated—the IvyGAP CT, CTmvp, CTpan, IT, and LE zone modules—were derived from the same IvyGAP transcriptomic atlas that provides our outcome data. The gene-set definitions and the ssGSEA scores on which we test radiomic association are therefore not independent sources of information, and any association involving these five modules is a consistency check on the zone definition, not a biological discovery. This circularity affects interpretation in three concrete ways. First, the IvyGAP CTpan module showed positive nested-CV R^2^ (0.133) and a significant composition-baseline effect (Spearman rho = 0.579, *p* = 0.001) but did not survive the conservative 24-pathway FDR correction; we treat it as uninterpretable rather than as a borderline finding. Second, the zero-signal result for the four other zone modules (Section 4.3) is most safely read as consistent with mapping attenuation, since a substantive test of zone-module detectability would require externally defined gene sets. Third, the 15 Hallmark and 4 Neftel signatures, all wholly external to IvyGAP, remain the only gene sets from which independent radiogenomic inference can be drawn in the present data. All substantive biological claims in this manuscript are therefore restricted to these 19 external signatures, and the five IvyGAP modules are retained only for completeness of reporting.

### 4.5. Limitations

This study has several important limitations.

Firstly, the zone-to-subcompartment mapping is biologically approximate rather than spatially precise, with weak correlations (mean r = 0.242), as reported by Park et al. [8]. All results should be interpreted within this context. Although a classical measurement error attenuation model suggests true effect sizes could be larger, its applicability to categorical zone-to-subcompartment mapping is uncertain and cannot be verified with the current data. The study is severely underpowered for the scope of modeling. With N = 28 matched patients against a Riley criterion of N = 240 for *p* = 5, R^2^ = 0.20, and shrinkage S ≥ 0.90, predictive performance estimates from LOPO-CV are intrinsically unstable: feature selection inside each fold is noisy, bootstrap confidence intervals are wide, and point estimates of R^2^_cv_ may vary substantially under small perturbations of the dataset. All predictive results must therefore be regarded as exploratory rather than as validated performance estimates, the study is hypothesis-generating rather than definitive, and the possibility of false-positive associations cannot be excluded. Additionally, there is no external validation cohort because IvyGAP is the only dataset that combines zone-level RNA-seq with matched MRI, preventing independent replication. The MRI data were obtained from a single institution, which may limit generalizability across scanners, though this also reduces batch effects. BraTS-style segmentations are radiological rather than biological boundaries. In the associational analysis, feature pre-selection on the full dataset may inflate significance, though nested cross-validation results help mitigate this concern. The R^2^ decomposition now separates radiomic increments from subcompartment effects, offering a clearer interpretation. Since IvyGAP zone modules are scored on the same expression data used for their derivation, circularity affects certain modules like CTpan, which showed positive R^2^_cv_ but should be interpreted cautiously. The LMD samples represent microscopic tissue volumes—roughly 500–2000 cells—covering less than 0.01% of the subcompartment, across centimeters, which introduces a cross-scale gap between the transcriptomic measurements and radiomic features that integrate signals over full subcompartment volumes. FPKM normalization was used instead of TPM or raw counts, with rank-based ssGSEA partially mitigating this. Permutation *p*-values for the LMM were around 0.05, warranting cautious interpretation, while the nested CV permutation *p*-value of 0.008 offers a more rigorous bound; the true significance likely lies between these values (see Section 4). Treatment confounding cannot be fully assessed. Bevacizumab (anti-VEGF) alters enhancement patterns and suppresses angiogenic transcriptomic programs; however, its use is unlikely in this cohort, as 27 of 28 patients had primary tumors with no prior anti-angiogenic therapy. Dexamethasone, routinely administered perioperatively in GBM, suppresses NF-kB-mediated inflammatory gene expression while simultaneously reducing peritumoral edema and T2/FLAIR signal; this dual suppression would attenuate both the transcriptomic and radiomic components of the inflammatory signal, biasing the observed association toward the null rather than inflating it. Temozolomide and radiation effects on the tumor microenvironment cannot be excluded but are less likely to create spurious radiomic–transcriptomic correlations in primary tumors imaged before adjuvant treatment. Dexamethasone use at the time of MRI is unknown from the IvyGAP metadata. Overall, the direction of potential treatment confounding is toward attenuation (conservative bias) rather than inflation of observed associations. Finally, WHO 2021 molecular markers and the top feature caps were not available or were sensitivity-tested.

### 4.6. Future Directions

If confirmed with spatially precise co-registration, several clinical application scenarios emerge. First, radiomic identification of inflammatory-enriched subregions could inform pre-operative molecular stratification, enabling selection of patients for immunotherapy trials targeting the tumor-associated macrophage compartment. Second, T2 texture features that track inflammatory response could serve as non-invasive longitudinal biomarkers for treatment response assessment, avoiding the need for repeat biopsy. Third, radiomic profiling of Angiogenesis-enriched regions could guide surgical planning by identifying subregions likely to harbor residual anti-angiogenic disease. Fourth, if radiomic–transcriptomic mapping is validated with spatial co-registration, dose painting strategies could target molecularly aggressive subregions during radiotherapy planning.

Beyond conventional MRI sequences used in this study (T1, T1-gadolinium, T2, FLAIR), advanced acquisition techniques offer complementary molecular information. Quantitative susceptibility mapping (QSM) captures iron deposition and calcification patterns associated with tumor grade and molecular subtype; Rui et al. [36] demonstrated that QSM-derived features combined with deep learning achieved high accuracy (AUC = 0.91) for glioma grading and molecular subtyping. Perfusion MRI (dynamic susceptibility contrast, dynamic contrast-enhanced) provides hemodynamic information, and diffusion-weighted imaging (apparent diffusion coefficient, diffusion kurtosis) captures microstructural properties not reflected in conventional radiomics. Future studies should evaluate whether combining conventional radiomic features with these advanced sequences improves the prediction of transcriptomic programs beyond the T2-dominated signal identified here.

More broadly, future studies should prioritize spatially co-registered datasets extending the Hu et al. [10] approach, multi-institutional harmonized radiomic extraction, and deep learning-derived features that may capture nonlinear relationships beyond hand-crafted IBSI features.

## 5. Conclusions

In this exploratory analysis of 28 patients with matched MRI radiomic features and zone-level RNA-seq data from the IvyGAP atlas, Inflammatory Response (R^2^_cv_ = 0.185, 95% CI [0.071, 0.355]) was the only pathway supported by both the nested-CV predictive analysis (FDR = 0.096) and the exploratory LMM (FDR = 0.024, ΔR^2^ = 0.214 beyond subcompartment effects), driven by T2-derived texture features. Angiogenesis (R^2^_cv_ = 0.209, 95% CI [0.028, 0.353]) reached significance only in the nested CV (FDR = 0.096) and was not corroborated by the LMM (FDR = 0.445); it is reported as a tentative signal requiring independent validation before any biological claim. The absence of signal for 21 of 24 pathways should not be read as evidence of biological inaccessibility: at N = 28 (vs. N ≈ 240 required), severe underpowering, attenuation from the non-spatial zone-to-subcompartment mapping (r^2^ ≈ 0.059), and methodological constraints each independently suffice to suppress real associations. Five of the 24 gene sets (the IvyGAP zone modules) are structurally non-independent from the outcome data and are retained only as internal-consistency checks; all substantive biological claims are restricted to the 19 external (Hallmark and Neftel) signatures. All reported associations are zone-approximate; validation in larger cohorts with spatially precise co-registration is essential before any clinical translation.

## Figures and Tables

**Table 1:** Zone-to-subcompartment mapping with biological rationale and empirical support.

IvyGAP Zone	MRI Subcompartment	Biological Rationale	Park et al. [8] Correlation
CT + CTmvp ^1^	Enhancing Tumor (ET)	Viable proliferating core and active Angiogenesis are the principal sources of gadolinium enhancement	r = 0.238 (CT-ET), r = 0.195 (CTmvp-ET)
CTpan	Non-Enhancing Tumor (NET)	Pseudopalisading necrosis regions are predominantly located within the non-enhancing tumor core	r = 0.241
IT + LE	Peritumoral Edema (ED)	Infiltrating tumor and leading edge extend into the FLAIR-hyperintense peritumoral zone	r = 0.294 (mean IT/LE-ED)

^1^ CTmvp (microvascular proliferation) samples are transcriptomically heterogeneous and may share features with CTpan. Sensitivity analysis S1a tests the robustness of all results when CTmvp is excluded from the ET subcompartment.

**Table 2:** Nested cross-validation results for pathways with R^2^_cv_ > 0.

Pathway	R^2^_cv_	95% CI ^e^	MAE	Spearman Rho	Stable Features (>50% Folds)	Nested Perm *p*	FDR (24) ^1^
Angiogenesis	0.209	[0.028, 0.353]	0.702	0.581	5	0.006	0.096
Inflammatory Response ^c^	0.185	[0.071, 0.355]	0.674	0.524	5	0.008	0.096
IvyGAP CTpan module ^d^	0.133	[−0.079, 0.350]	0.740	0.348	4	0.013	0.104

^1^ Two FDR correction approaches are reported for transparency. The conservative approach applies BH-FDR across all 24 pathways, assigning *p* = 1.0 to 21 pathways with R^2^_cv_ ≤ 0 (FDR = 0.096 for Angiogenesis and Inflammatory Response; Table S10). The standard approach applies BH-FDR restricted to the three pathways with R^2^_cv_ > 0 (FDR = 0.012 for both Angiogenesis and Inflammatory Response). We lead with the conservative correction to avoid selection bias, but the standard correction is provided as a transparency comparison. ^c^ Designated as primary based on convergent LMM evidence (Section 3.4). Both analyses use the same outcome data and are not independent; this represents consistency across different statistical models on the same dataset, not independent replication. ^d^ CI crosses zero; gene module scored on the same expression data from which it was derived (see Limitation 7); composition baseline test significant (*p* = 0.001). Interpret with caution. ^e^ Bootstrap CIs (B = 1000) condition on fitted predictions and capture metric sampling variability only; see Section 2.6 for interpretation.

**Table 3:** Linear mixed-effects model results for all 12 tested pathways, ordered by FDR. R^2^_m_ (null) = subcompartment + random intercept only; ΔR^2^_m_ = radiomic increment.

Pathway	Category	k	R^2^_m_ (Null)	R^2^_m_ (Full)	ΔR^2^_m_	R^2^_c_	LRT *p*	FDR (24)
Inflammatory Response	Hallmark	5	0.170	0.384	0.214	0.687	0.001	0.024 *
Angiogenesis	Hallmark	1	0.425	0.459	0.034	0.582	0.053	0.445
Hypoxia	Hallmark	5	0.837	0.847	0.010	0.918	0.085	0.445
*P53* Pathway	Hallmark	5	0.470	0.533	0.063	0.716	0.101	0.445
Glycolysis	Hallmark	5	0.789	0.804	0.015	0.849	0.112	0.445
mTORC1 Signaling	Hallmark	3	0.671	0.694	0.024	0.709	0.133	0.445
Neftel MES	Neftel	2	0.546	0.562	0.016	0.699	0.133	0.445
Complement	Hallmark	5	0.059	0.208	0.149	0.462	0.148	0.445
EMT	Hallmark	5	0.339	0.409	0.070	0.589	0.176	0.469
TNFA/NF-kB	Hallmark	5	0.580	0.610	0.030	0.703	0.352	0.845
IvyGAP CTpan Module	IvyGAP	5	0.906	0.902	−0.004	0.922	0.773	1.000
Oxidative Phosphorylation	Hallmark	3	0.352	0.344	−0.008	0.557	0.800	1.000

* FDR < 0.05 across all 24 pathways (BH correction). Because the Nakagawa–Schielzeth R^2^ is not strictly additive in mixed models, ΔR^2^_m_ values are approximate; negative values (CTpan, OxPhos) reflect variance repartitioning.

## Data Availability

The IvyGAP RNA-seq data are publicly available from the Allen Institute for Brain Science (https://glioblastoma.alleninstitute.org/, accessed on 15 January 2026). The IVYGAP-RADIOMICS radiomic feature set is publicly available from The Cancer Imaging Archive (TCIA; https://doi.org/10.7937/9j41-7d44, accessed on 15 January 2026). Analysis code is available at https://github.com/dpiccolomd/RADIOMAP-IvyGAP (accessed on 15 January 2026).

## Supplementary Materials

The following supporting information can be downloaded at https://www.mdpi.com/article/10.3390/cancers18081293/s1, Methods S1: Technical Details of Nested Cross-Validation; Figure S1: Exploratory linear mixed-effects model (LMM) results for all 12 pathways that retained radiomic features after univariate screening. Each point represents the marginal R^2^ (variance explained by fixed effects only) for a given pathway. Horizontal lines extend from zero to the point estimate. Red points indicate pathways reaching FDR < 0.05 after Benjamini–Hochberg correction across 12 tests; gray points indicate non-significant pathways (NS). The dashed vertical line marks R^2^_marginal_ = 0. Only the Inflammatory Response pathway reached significance (FDR = 0.012). Features were pre-selected on the full dataset; these results carry optimistic bias and should be interpreted alongside the nested cross-validation analysis (Figure 2). LMM = linear mixed-effects model; FDR = false discovery rate; R^2^_marginal_ = Nakagawa marginal R^2^; Figure S2: Standardized coefficients for the five radiomic features in the Inflammatory Response LMM. Points represent standardized beta coefficients; horizontal error bars indicate 95% confidence intervals. The dashed vertical line marks zero. Red points denote features significant after Holm correction (*p* < 0.05); gray points denote non-significant features (NS). All five features are T2-derived. R^2^_m_ = 0.384, FDR = 0.012. LMM = linear mixed-effects model; GLSZM = Gray-Level Size Zone Matrix; CoLIAGe = Co-occurrence of Local Anisotropic Gradient Orientations; Figure S3: Residual diagnostics for the Inflammatory Response LMM. (A) Normal Q-Q plot of standardized residuals. Blue points represent individual observations; the red line indicates the theoretical normal distribution. (B) Cook’s distance for each observation. Red bars indicate observations exceeding the conventional influence threshold of 4/n = 0.080 (dashed red horizontal line); blue bars indicate non-influential observations. One observation exceeded Cook’s D = 1.0. (C) Residuals versus fitted values, colored by MRI subcompartment: blue = enhancing tumor (ET), orange = non-enhancing tumor (NET), green = peritumoral edema (ED). The dashed horizontal line marks zero. No systematic pattern is evident. LMM = linear mixed-effects model; Figure S4: Permutation null distribution for the Inflammatory Response LMM. The histogram shows the distribution of likelihood ratio test (LRT) chi-squared statistics obtained from 1000 permutations of the pathway enrichment scores. The dashed red vertical line indicates the observed LRT chi-squared = 20.53 (permutation *p* = 0.055). LMM = linear mixed-effects model; LRT = likelihood ratio test; Figure S5: Sensitivity analysis for the Inflammatory Response LMM across seven analysis variants. Each point represents the LRT *p*-value for a given variant, plotted as −log_10_(*p*). Red points indicate *p* < 0.05; the gray point indicates *p* ≥ 0.05. The dashed vertical line marks *p* = 0.05. Variants: Primary = default analysis; S1a = ET mapped to Cellular Tumor only (excluding MVP); S1b = conservative zone mapping; S2 = patients with ≥2 subcompartments only; S3 = median aggregation (instead of mean); S7 = global standardization; S8 = Kenward-Roger denominator degrees of freedom. Six of seven variants retained significance. LMM = linear mixed-effects model; LRT = likelihood ratio test; ET = enhancing tumor; MVP = microvascular proliferation; Figure S6: Heatmap of Spearman correlations between the top 30 radiomic features and 12 pathway enrichment scores that retained features after univariate screening. Color scale represents Spearman rho (red = positive correlation, blue = negative correlation). Hierarchical clustering dendrograms (Ward’s method) are applied to both rows (features) and columns (pathways). FDR significance is annotated in the color bar (<0.05 vs. ≥0.05). Feature names follow the convention: MRI sequence, texture class, extraction parameters. FDR = false discovery rate; Figure S7: Volcano plot of univariate radiomic-transcriptomic associations for the Inflammatory Response pathway. Each point represents one of 597 candidate radiomic features after unsupervised filtering. The *x*-axis shows the maximum absolute Spearman rho across the three MRI subcompartments; the *y*-axis shows −log_10_(FDR). Red points indicate FDR < 0.05 (1 feature); orange points indicate FDR < 0.10 (24 features); gray points indicate non-significant features. Dashed horizontal lines mark the FDR < 0.05 and FDR < 0.10 thresholds. FDR = false discovery rate; Figure S8: Cohort structure and data availability. Heatmap showing the number of zone-aggregated RNA-seq samples per patient (rows) per MRI subcompartment (columns). Color intensity is proportional to sample count (white = 0, dark blue = 8); numbers within cells indicate exact counts. All 28 patients with matched IvyGAP transcriptomic and IVYGAP-RADIOMICS data are shown. ET = enhancing tumor (mapped from Cellular Tumor and Microvascular Proliferation IvyGAP zones); NET = non-enhancing tumor (mapped from Pseudopalisading Necrosis); ED = peritumoral edema (mapped from Infiltrating Tumor and Leading Edge); Figure S9: Unsupervised radiomic feature reduction pipeline. Bar chart showing progressive dimensionality reduction across four stages: 3920 raw IBSI-compliant features per subcompartment, 3860 after near-zero-variance (NZV) filtering, 597 after Spearman correlation pruning (|r| > 0.90, retaining one feature per correlated cluster), and 29 unique features entering final models after pathway-specific univariate screening. NZV = near-zero-variance; IBSI = Image Biomarker Standardization Initiative; Figure S10: Flowchart of the radiomic feature reduction pipeline. Stage 1 (near-zero-variance filtering) and Stage 2 (pairwise Spearman correlation pruning, |r| > 0.90) are unsupervised. Stage 3 (per-pathway univariate screening, FDR < 0.10, top 5) is supervised: applied on the full dataset for the exploratory LMM (Section 2.5) and independently inside each LOPO fold for the primary nested CV (Section 2.6); Table S1: Nested cross-validation results for all 24 pathways (R^2^_cv_, MAE, stable features). Pathways are ordered by descending R^2^_cv_. Feature selection was performed independently inside each LOPO fold (no data leakage). Stable features = features selected in >50% of folds; Table S2: Status of all 24 pathways in the associational analysis (12 tested in LMM, 12 with zero features passing univariate filter, 1 FDR-significant). Pathways with zero univariate features were not tested in the LMM; Table S3: Full sensitivity analysis results for the Inflammatory Response pathway across all analysis variants. R^2^_m_ = marginal R^2^ (fixed effects only); LRT *p* = likelihood ratio test *p*-value comparing full model (radiomic features + subcompartment) to null model (subcompartment only). All variants use the same five radiomic features except where noted; Table S4: Gene set pairwise Jaccard similarity matrix (24 × 24). Values represent the Jaccard index (intersection/union of gene members) between gene sets. Higher values indicate greater overlap. Most pairs show minimal overlap (J < 0.10), confirming that the 24 gene sets capture largely distinct biological programs. Notable exceptions: IvyGAP IT and LE modules (J = 0.653); *E2F* Targets and G2M Checkpoint (J = 0.223); Glycolysis and Hypoxia (J = 0.194); IvyGAP CTpan and Hypoxia (J = 0.146); Inflammatory Response and TNFA/NF-kB (J = 0.146); Table S5: Legacy pre-screened Elastic Net results compared with nested CV results. The legacy analysis pre-selected features on the full dataset before LOPO-CV, introducing data leakage. Only pathways with R^2^_cv_ > 0 in either analysis are shown; Table S6: Feature lookup table mapping radiomic feature indices to full IBSI names, MRI sequence, and feature type. All 89 unique radiomic features that entered any model across all 24 pathways are listed; Table S7: Clinical covariate adjustment results for the Inflammatory Response pathway (sensitivity analysis S9; Section 2.9). Progressive covariate adjustment demonstrating that radiomic features remain significant after accounting for age, *MGMT* methylation status, and molecular subtype. LRT tests the contribution of the five radiomic features above the covariates included in each model; Table S8: CLEAR (CheckList for EvaluAtion of Radiomics research) compliance table. Self-assessment following CLEAR v1.0 [32]; Table S9: Top radiomic features for the Angiogenesis pathway, identified by stability selection across 28 LOPO folds. Features selected in >50% of folds are considered stable; Table S10: BH-FDR-corrected nested CV permutation *p*-values for all 24 pathways. Pathways with R^2^_cv_ ≤ 0 were assigned *p* = 1.0 before FDR correction (conservative approach); Table S11: Hyperparameter distributions (alpha, lambda) across LOPO folds for the three pathways with positive predictive signal. Alpha was selected from a grid of 0.1 to 1.0 (step 0.1); lambda was selected using the 1-SE rule (lambda.1se) from inner 5-fold CV; Table S12: Top radiomic features for the Inflammatory Response pathway, identified by stability selection across 28 LOPO folds. Full IBSI feature names, fold selection counts, and stability percentages are provided. Features selected in >50% of folds are considered stable; Table S13: Coefficient-level results for the Inflammatory Response linear mixed-effects model. Standardized beta coefficients, standard errors, 95% confidence intervals, Type II Satterthwaite ANOVA F-values, and Holm-adjusted *p*-values for all five radiomic features and the subcompartment fixed effect. Model: R^2^_m_ = 0.384, R^2^_c_ = 0.687, LRT chi^2^ = 20.53, df = 5, *p* = 0.001.

## Abbreviations

The following abbreviations are used in this manuscript:

Abbreviation	Full Term
AC	Astrocyte-like
ADC	Apparent Diffusion Coefficient
BH	Benjamini–Hochberg
BraTS	Brain Tumor Segmentation
CaPTk	Cancer Imaging Phenomics Toolkit
CBV	Cerebral Blood Volume
CDKN2A/B	Cyclin-Dependent Kinase Inhibitor 2A/B
CI	Confidence Interval
CLEAR	CheckList for EvaluAtion of Radiomics research
CoLIAGe	Co-occurrence of Local Anisotropic Gradient Orientations
CT	Cellular Tumor
CTmvp	Cellular Tumor—Microvascular Proliferation
CTpan	Cellular Tumor—Pseudopalisading Necrosis
CV	Cross-Validation
ED	Peritumoral Edema
EMT	Epithelial–Mesenchymal Transition
ET	Enhancing Tumor
FDR	False Discovery Rate
FLAIR	Fluid-Attenuated Inversion Recovery
FPKM	Fragments Per Kilobase of Transcript Per Million Mapped Reads
GBM	Glioblastoma
GLCM	Gray-Level Co-occurrence Matrix
GLRLM	Gray-Level Run Length Matrix
GLSZM	Gray-Level Size Zone Matrix
GSEA	Gene Set Enrichment Analysis
IBSI	Image Biomarker Standardization Initiative
ICC	Intraclass Correlation Coefficient
*IDH*	Isocitrate Dehydrogenase
IFN	Interferon
IL-6	Interleukin-6
IT	Infiltrating Tumor
IvyGAP	Ivy Glioblastoma Atlas Project
JAK	Janus Kinase
KPS	Karnofsky Performance Status
LBP	Local Binary Pattern
LE	Leading Edge
LMD	Laser Microdissection
LMM	Linear Mixed-Effects Model
LOPO-CV	Leave-One-Patient-Out Cross-Validation
LRT	Likelihood Ratio Test
MAE	Mean Absolute Error
MES	Mesenchymal-like
*MGMT*	O^6^-Methylguanine-DNA Methyltransferase
MRI	Magnetic Resonance Imaging
MSigDB	Molecular Signatures Database
mTORC1	Mechanistic Target of Rapamycin Complex 1
NET	Non-Enhancing Tumor
NF-κB	Nuclear Factor Kappa B
NGTDM	Neighborhood Grey-Tone Difference Matrix
NPC	Neural Progenitor-like
NZV	Near-Zero Variance
OPC	Oligodendrocyte Progenitor-like
RNA-seq	RNA Sequencing
SD	Standard Deviation
SHAP	SHapley Additive exPlanations
ssGSEA	Single-Sample Gene Set Enrichment Analysis
STAT3	Signal Transducer and Activator of Transcription 3
TCIA	The Cancer Imaging Archive
TERT	Telomerase Reverse Transcriptase
TNF-α	Tumor Necrosis Factor Alpha
TPM	Transcripts Per Million
VEGF	Vascular Endothelial Growth Factor
WHO	World Health Organization
