# Supplementary material for: Radiomic Features of MRI Subcompartments Associate with Angiogenic and Inflammatory Transcriptomic Programs in Glioblastoma: An IvyGAP Exploratory Analysis
Source: Cancers (Basel). 2026 Apr 19;18(8):1293. doi: 10.3390/cancers18081293 (PMC13114458; doi:10.3390/cancers18081293)
Supplement: Supplementary file 1 [file cancers-18-01293-s001.zip › cancers-4212516-supplementary.pdf]

SUPPLEMENTARY MATERIALS

Supplementary Methods S1. Technical Details of Nested Cross-Validation

Bootstrap confidence intervals. Uncertainty around R^2_{cv} was quantified via nonparametric bootstrap with B = 1000 resamples. Each bootstrap resample condition on the fitted predictions from the full nested LOPO-CV procedure without refitting the complete pipeline. Consequently, these confidence intervals capture only the sampling variability of the performance metric, not the variability introduced by feature selection or hyperparameter tuning across folds. They should not be interpreted as reflecting the full uncertainty of the predictive signal. The nested permutation p-values (Section 2.7), which re-execute the entire pipeline — including feature selection — under the null hypothesis, remain the primary inferential tool.

Hyperparameter selection. The Elastic Net mixing parameter alpha was selected from a grid of {0.1, 0.2, 0.3, 0.4, 0.5, 0.6, 0.7, 0.8, 0.9, 1.0}. The regularization parameter lambda was selected using the conservative one-standard-error rule (lambda.1se) from an inner 5-fold cross-validation on each training set of 27 patients. Inner 5-fold CV on such a small training set produces noisy hyperparameter selection, and alpha and lambda distributions across the 28 outer LOPO folds are reported in Supplementary Table S11 to document this variability. Despite this noise, feature stability across folds (Figure 3 for Inflammatory Response; Supplementary Table S9 for Angiogenesis) was high, indicating that the feature-level signal is more robust than the hyperparameter-level signal.

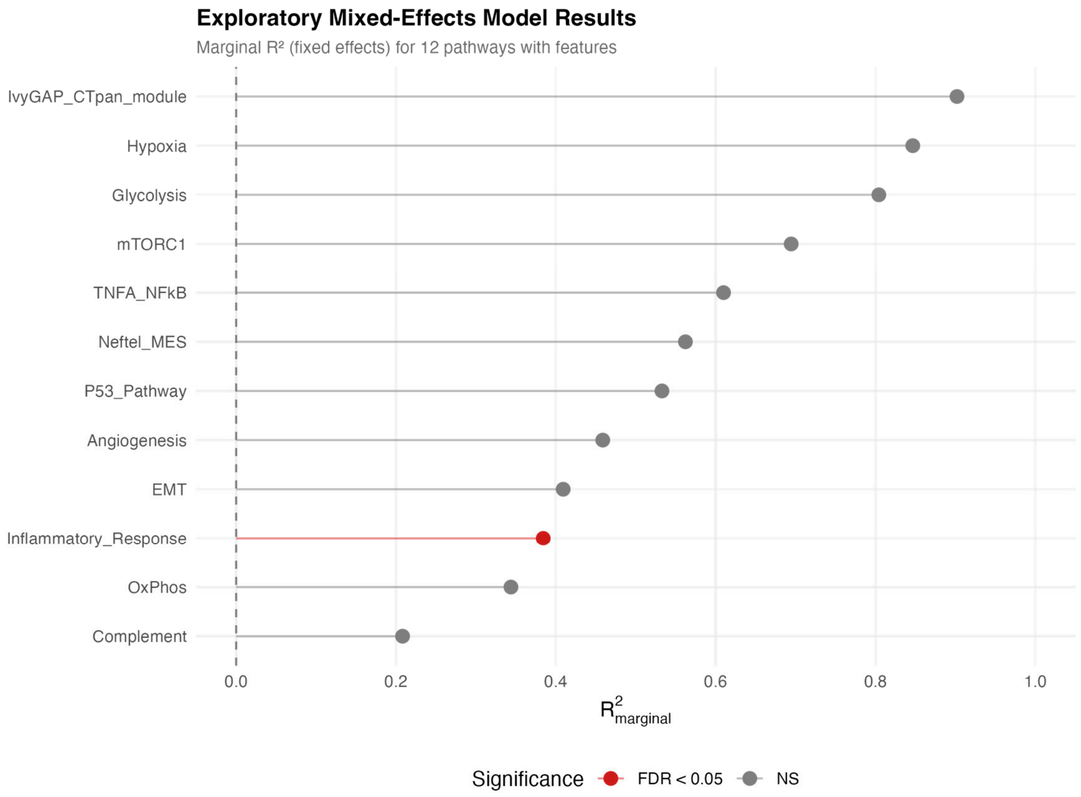

Figure S1. Exploratory linear mixed-effects model (LMM) results for all 12 pathways that retained radiomic features after univariate screening. Each point represents the marginal R^2 (variance explained by fixed effects only) for a given pathway. Horizontal lines extend from zero to the point estimate. Red points indicate pathways reaching FDR < 0.05 after Benjamini-Hochberg correction across 12 tests; gray points indicate non-significant pathways (NS). The dashed vertical line marks $R^2_{marginal} = 0$. Only the Inflammatory Response pathway reached significance (FDR = 0.012). Features were pre-selected on the full dataset; these results carry optimistic bias and should be interpreted alongside the nested cross-validation analysis (Figure 2). LMM = linear mixed-effects model; FDR = false discovery rate; $R^2_{marginal}$ = Nakagawa marginal R^2.

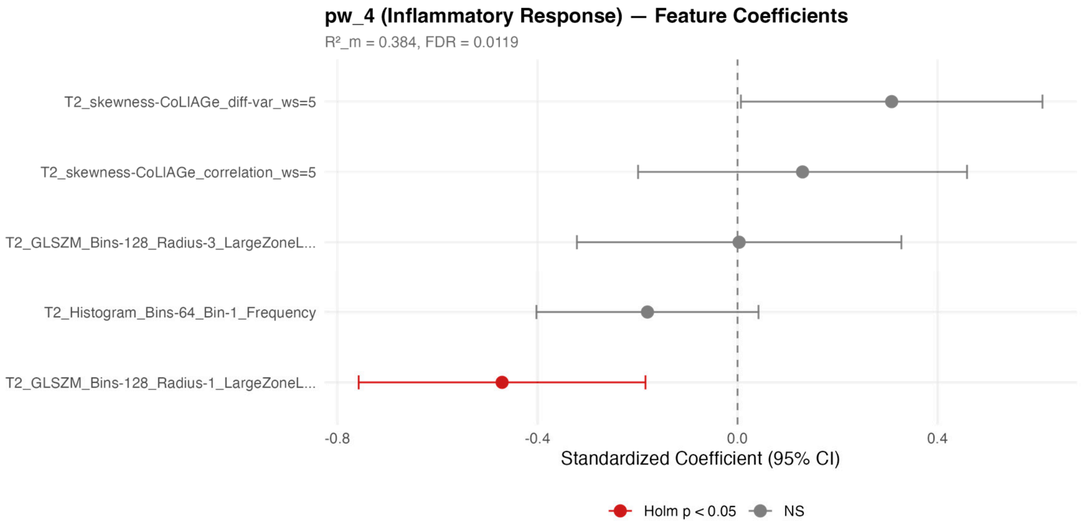

Figure S2. Standardized coefficients for the five radiomic features in the Inflammatory Response LMM. Points represent standardized beta coefficients; horizontal error bars indicate 95% confidence intervals. The dashed vertical line marks zero. Red points denote features significant after Holm correction ($p < 0.05$); gray points denote non-significant features (NS). All five features are T2-derived. $R^2_m = 0.384$, FDR = 0.012. LMM = linear mixed-effects model; GLSZM = Gray-Level Size Zone Matrix; CoLIAGe = Co-occurrence of Local Anisotropic Gradient Orientations.

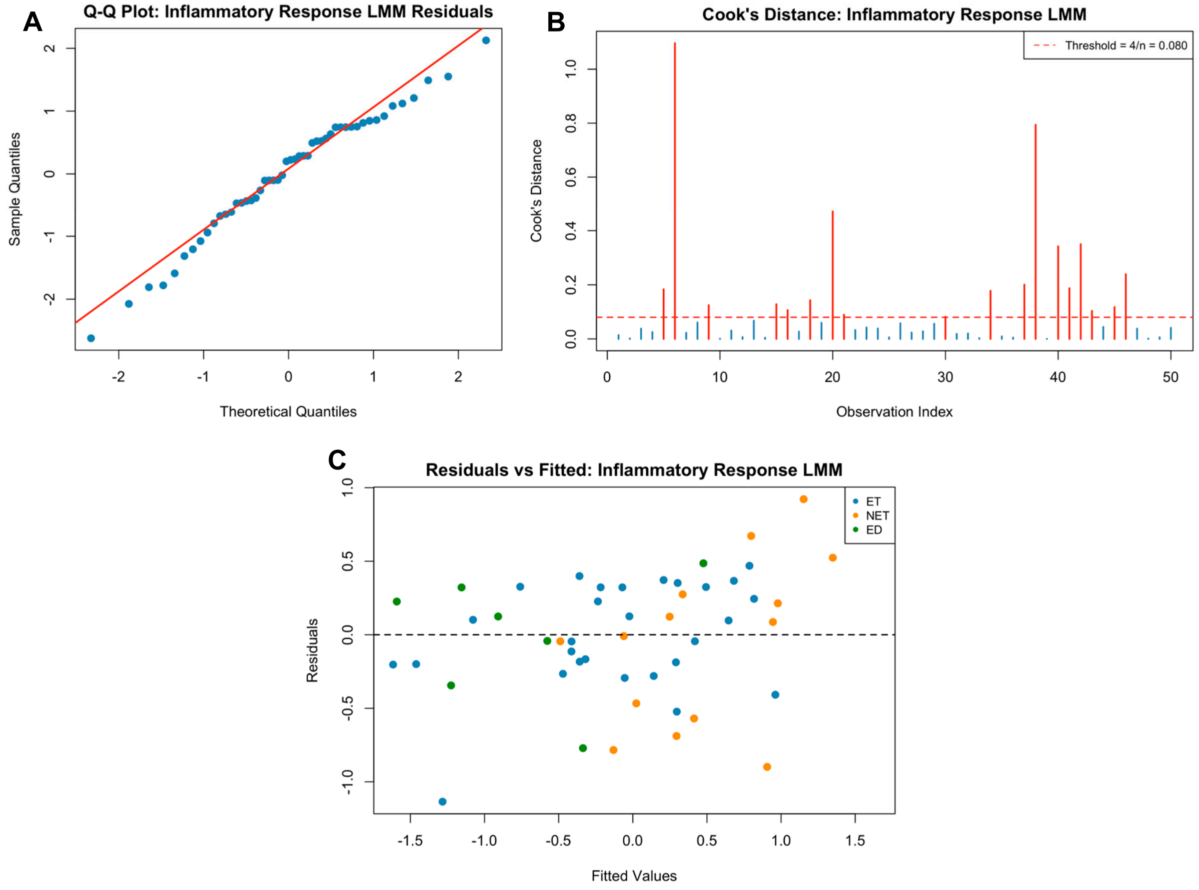

Figure S3. Residual diagnostics for the Inflammatory Response LMM. **(A)** Normal Q-Q plot of standardized residuals. Blue points represent individual observations; the red line indicates the theoretical normal distribution. **(B)** Cook's distance for each observation. Red bars indicate observations exceeding the conventional influence threshold of 4/n = 0.080 (dashed red horizontal line); blue bars indicate non-influential observations. One observation exceeded Cook's D = 1.0. **(C)** Residuals versus fitted values, colored by MRI subcompartment: blue = enhancing tumor (ET), orange = non-enhancing tumor (NET), green = peritumoral edema (ED). The dashed horizontal line marks zero. No systematic pattern is evident. LMM = linear mixed-effects model.

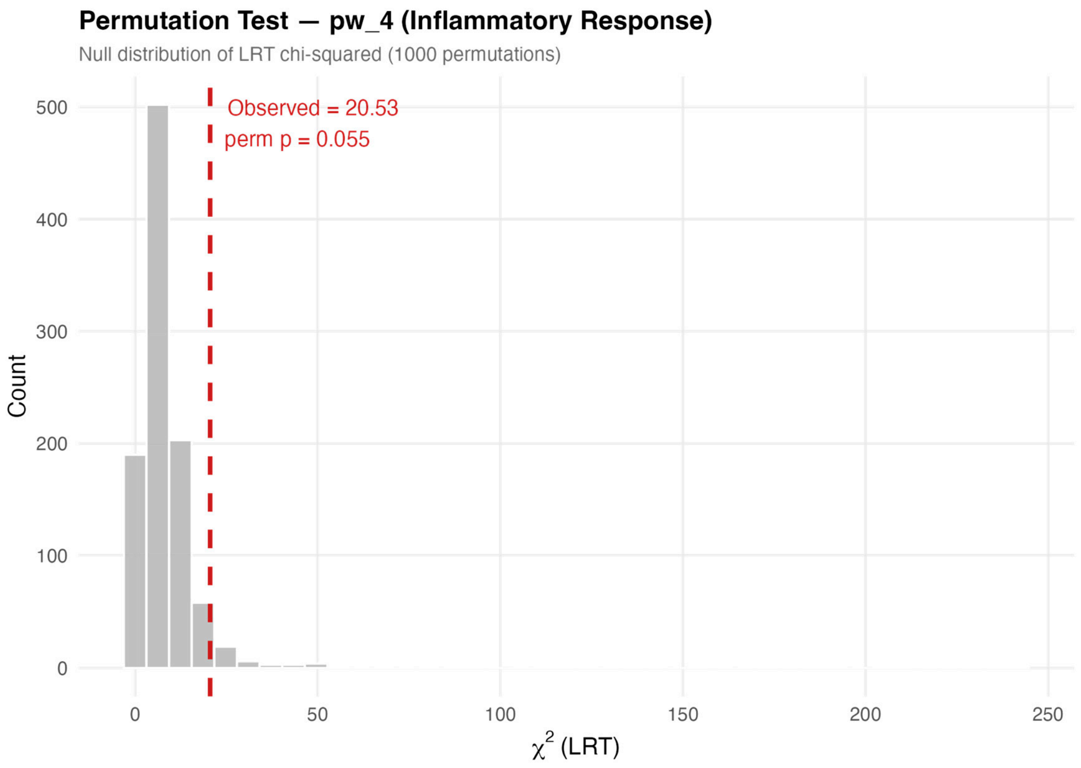

Figure S4. Permutation null distribution for the Inflammatory Response LMM. The histogram shows the distribution of likelihood ratio test (LRT) chi-squared statistics obtained from 1000 permutations of the pathway enrichment scores. The dashed red vertical line indicates the observed LRT chi-squared = 20.53 (permutation p = 0.055). LMM = linear mixed-effects model; LRT = likelihood ratio test.

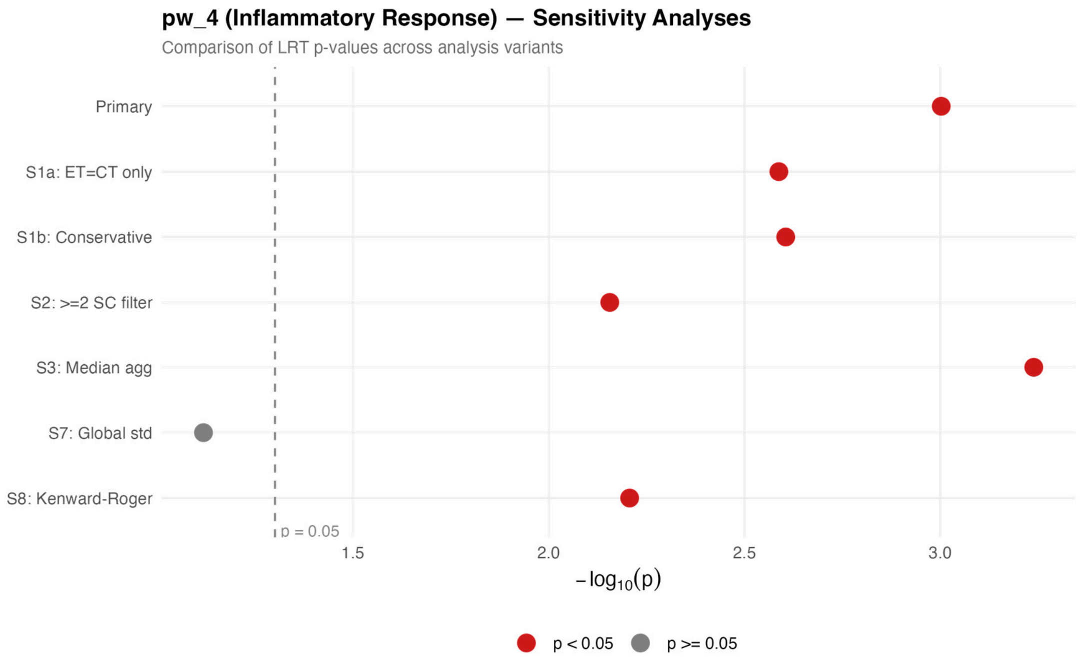

Figure S5. Sensitivity analysis for the Inflammatory Response LMM across seven analysis variants. Each point represents the LRT p-value for a given variant, plotted as $-\log_{10}(p)$. Red points indicate $p < 0.05$; the gray point indicates $p >= 0.05$. The dashed vertical line marks $p = 0.05$. Variants: Primary = default analysis; S1a = ET mapped to Cellular Tumor only (excluding MVP); S1b = conservative zone mapping; S2 = patients with >= 2 subcompartments only; S3 = median aggregation (instead of mean); S7 = global standardization; S8 = Kenward-Roger denominator degrees of freedom. Six of seven variants retained significance. LMM = linear mixed-effects model; LRT = likelihood ratio test; ET = enhancing tumor; MVP = microvascular proliferation.

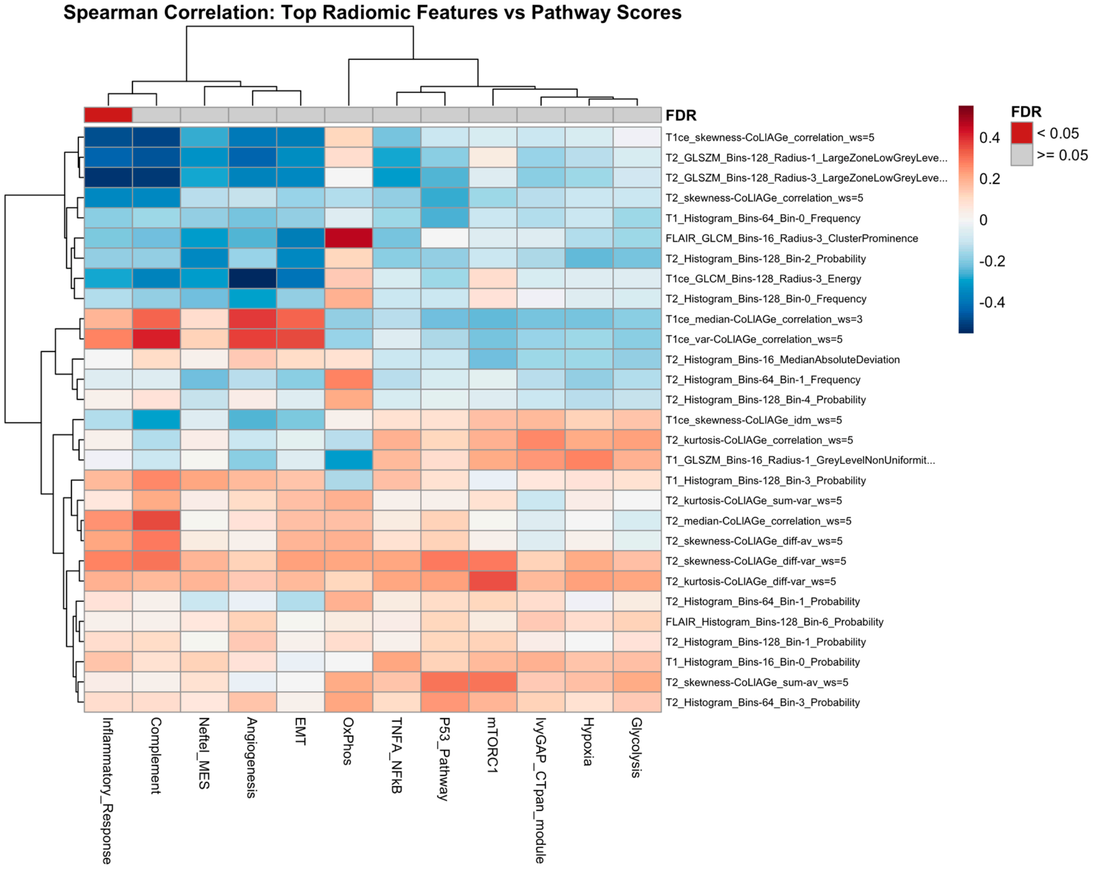

Figure S6. Heatmap of Spearman correlations between the top 30 radiomic features and 12 pathway enrichment scores that retained features after univariate screening. Color scale represents Spearman rho (red = positive correlation, blue = negative correlation). Hierarchical clustering dendrograms (Ward's method) are applied to both rows (features) and columns (pathways). FDR significance is annotated in the color bar (< 0.05 vs. >= 0.05). Feature names follow the convention: MRI sequence, texture class, extraction parameters. FDR = false discovery rate.

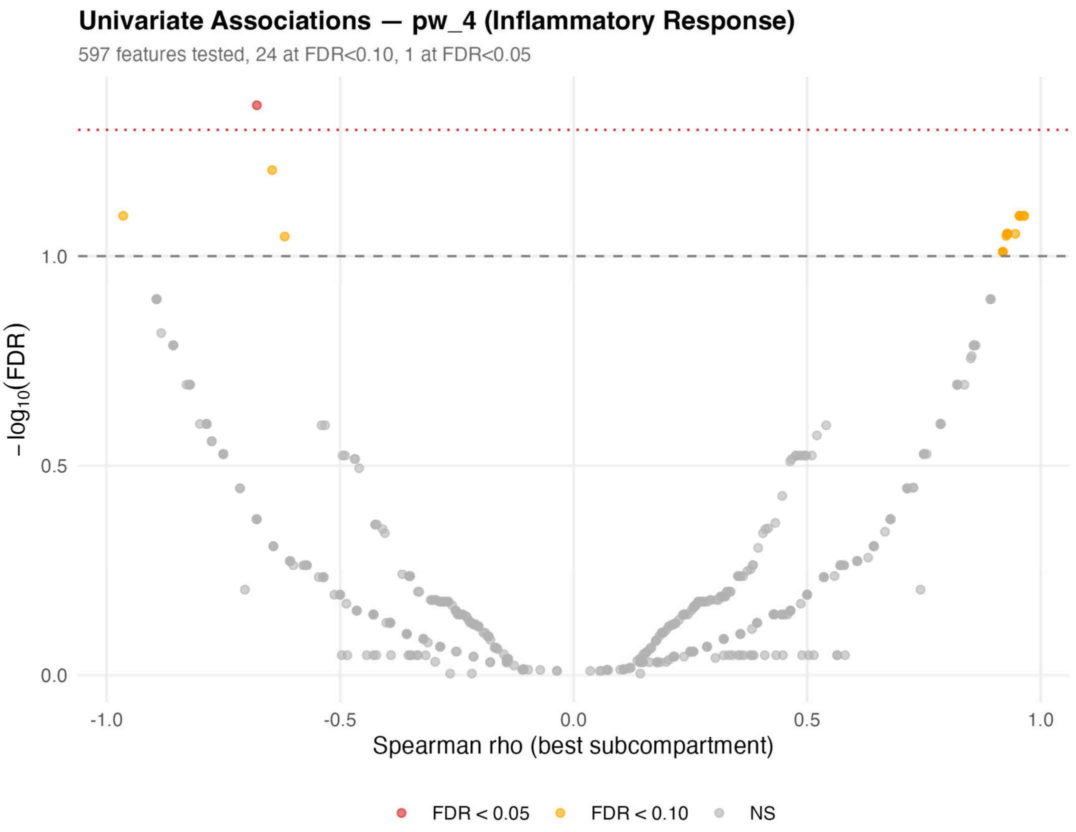

Figure S7. Volcano plot of univariate radiomic-transcriptomic associations for the Inflammatory Response pathway. Each point represents one of 597 candidate radiomic features after unsupervised filtering. The x-axis shows the maximum absolute Spearman rho across the three MRI subcompartments; the y-axis shows $-\log_{10}$(FDR). Red points indicate FDR < 0.05 (1 feature); orange points indicate FDR < 0.10 (24 features); gray points indicate non-significant features. Dashed horizontal lines mark the FDR < 0.05 and FDR < 0.10 thresholds. FDR = false discovery rate.

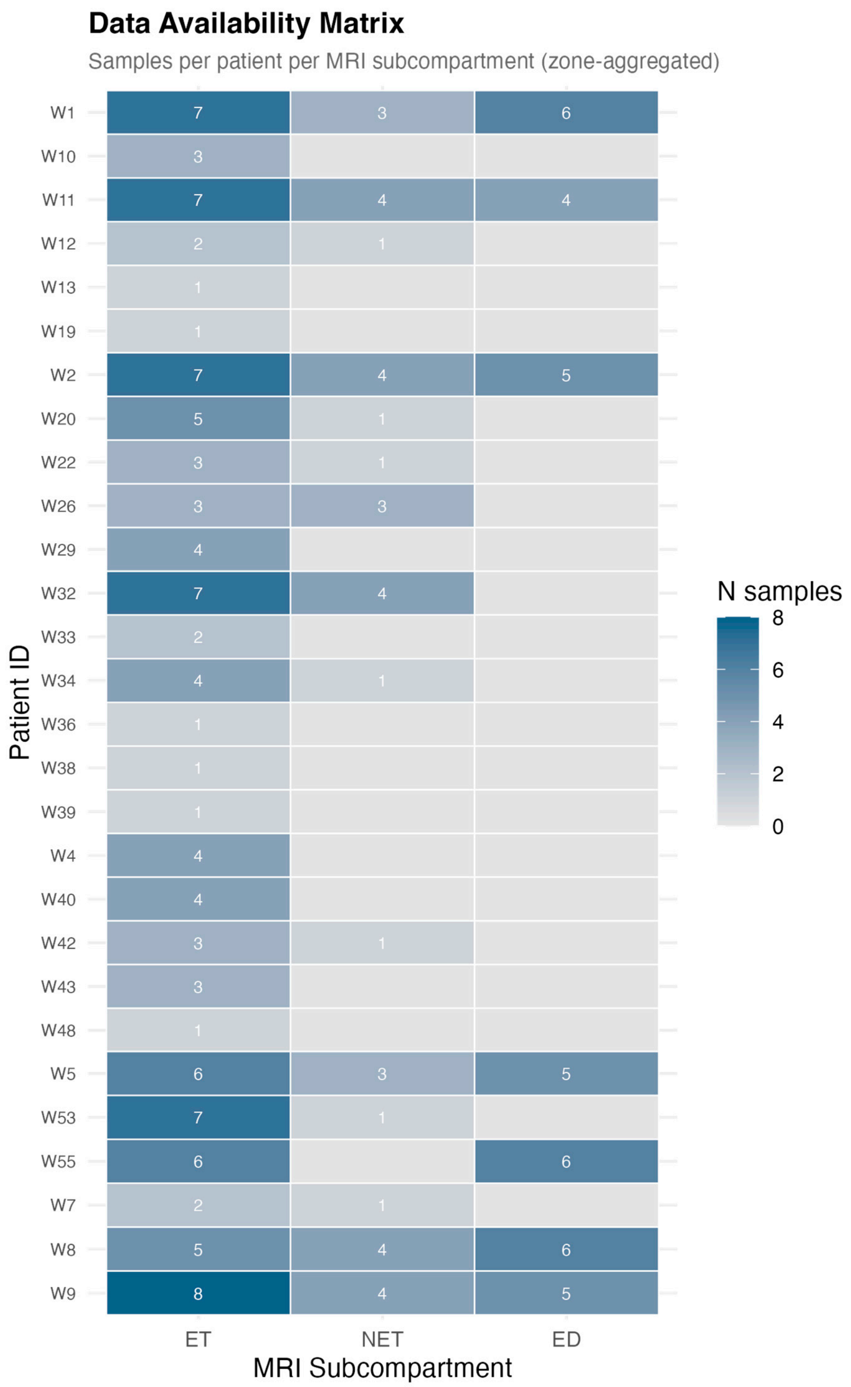

Figure S8. Cohort structure and data availability. Heatmap showing the number of zone-aggregated RNA-seq samples per patient (rows) per MRI subcompartment (columns). Color intensity is proportional to sample count (white = 0, dark blue = 8); numbers within cells indicate exact counts. All 28 patients with matched IvyGAP transcriptomic and IVYGAP-RADIOMICS data are shown. ET = enhancing tumor (mapped from Cellular Tumor and Microvascular Proliferation IvyGAP zones); NET = non-enhancing tumor (mapped from Pseudopalisading Necrosis); ED = peritumoral edema (mapped from Infiltrating Tumor and Leading Edge).

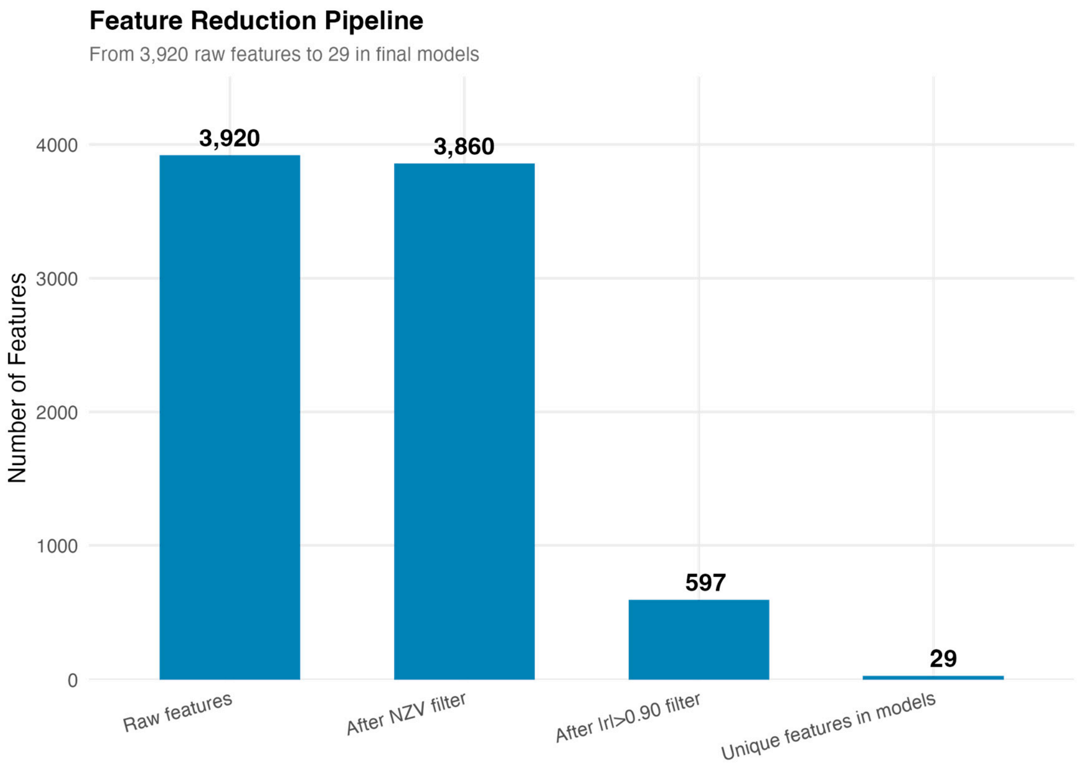

Figure S9. Unsupervised radiomic feature reduction pipeline. Bar chart showing progressive dimensionality reduction across four stages: 3920 raw IBSI-compliant features per subcompartment, 3860 after near-zero-variance (NZV) filtering, 597 after Spearman correlation pruning (|r| > 0.90, retaining one feature per correlated cluster), and 29 unique features entering final models after pathway-specific univariate screening. NZV = near-zero-variance; IBSI = Image Biomarker Standardisation Initiative.

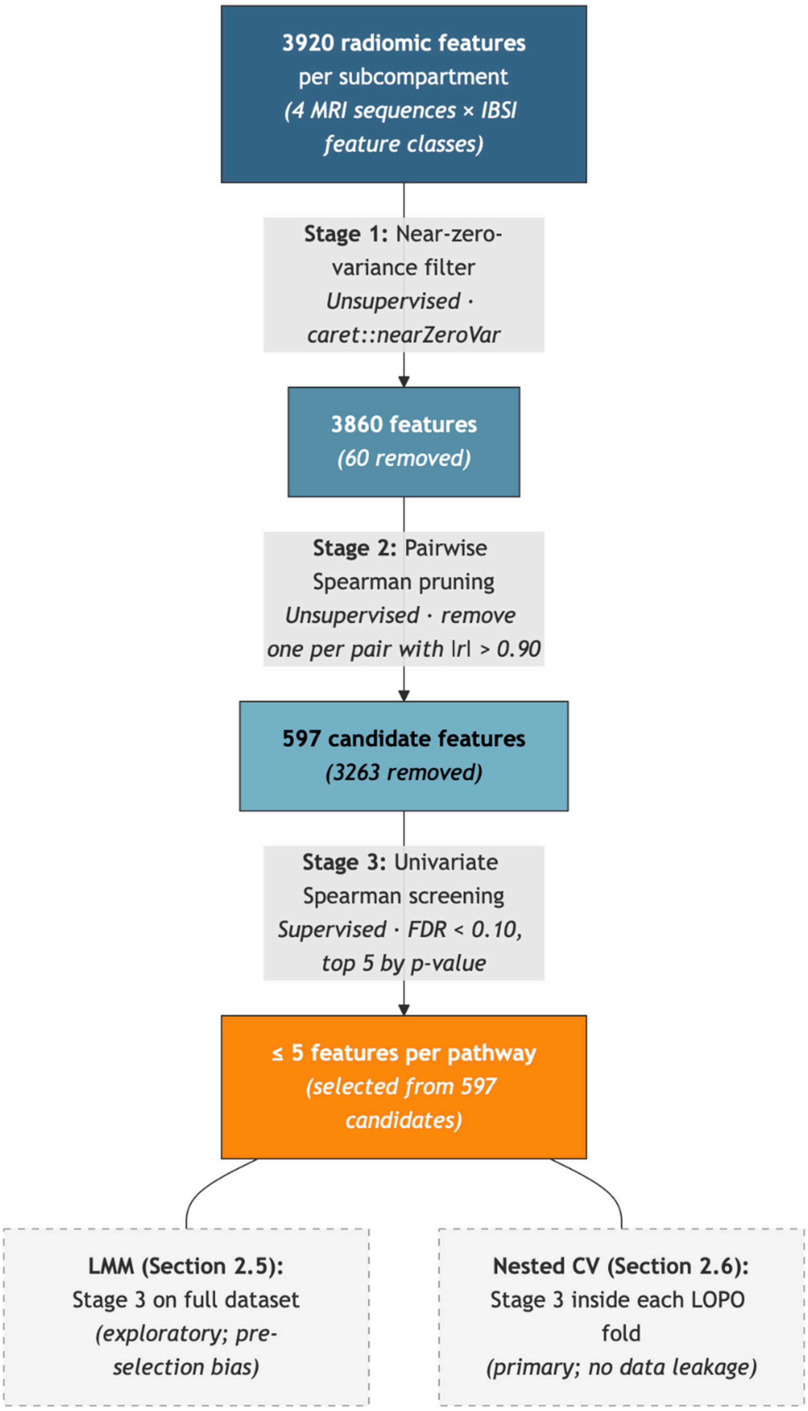

Figure S10. Flowchart of the radiomic feature reduction pipeline. Stage 1 (near-zero-variance filtering) and Stage 2 (pairwise Spearman correlation pruning, $|r| > 0.90$) are unsupervised. Stage 3 (per-pathway univariate screening, FDR < 0.10, top 5) is supervised: applied on the full dataset for the exploratory LMM (Section 2.5) and independently inside each LOPO fold for the primary nest-ed CV (Section 2.6).

Table S1. Nested cross-validation results for all 24 pathways (R^2_{cv}, MAE, stable features). Pathways are ordered by descending R^2_{cv}. Feature selection was performed independently inside each LOPO fold (no data leakage). Stable features = features selected in >50% of folds.

Pathway	R^2_{cv}	MAE	Spearman rho	Stable features	Median features/fold
Angiogenesis	0.209	0.702	0.581	5	5
Inflammatory Response	0.185	0.674	0.524	5	5
IvyGAP CTpan module	0.133	0.740	0.348	4	5
Complement	-0.004	0.680	0.194	5	5
EMT	-0.034	0.713	-0.607	1	1
IvyGAP IT module	-0.072	0.833	-0.784	1	1
P53 Pathway	-0.075	0.762	-1.000	0	0
E2F Targets	-0.075	0.823	-1.000	0	0
G2M Checkpoint	-0.075	0.831	-1.000	0	0
mTORC1 Signaling	-0.075	0.899	-1.000	0	0
Neftel NPC	-0.075	0.881	-1.000	0	0
IvyGAP CT module	-0.075	0.796	-1.000	0	0
MYC Targets V1	-0.075	0.753	-1.000	0	0
Oxidative Phosphorylation	-0.075	0.833	-1.000	0	0
Neftel AC	-0.075	0.789	-1.000	0	0
Neftel OPC	-0.086	0.820	-0.968	0	0
Glycolysis	-0.109	0.822	-1.000	0	0
TNFA/NF-kB	-0.113	0.795	-0.990	0	0
IvyGAP LE module	-0.114	0.828	-0.773	1	1
Hypoxia	-0.135	0.813	-0.443	0	0.5
Neftel MES	-0.146	0.790	-0.778	0	0
IL6/JAK/STAT3	-0.199	0.894	-0.748	3	3
IFN Gamma Response	-0.210	0.868	-0.806	0	0
IvyGAP CTmvp module	-0.274	0.893	-0.582	2	4

Table S2. Status of all 24 pathways in the associational analysis (12 tested in LMM, 12 with zero features passing univariate filter, 1 FDR-significant). Pathways with zero univariate features were not tested in the LMM.

Pathway	Category	Univariate features	Status	R^2_m	LRT p	FDR
Hypoxia	Hallmark	46	Tested in LMM	0.847	0.085	0.223
Angiogenesis	Hallmark	1	Tested in LMM	0.459	0.053	0.223
EMT	Hallmark	21	Tested in LMM	0.409	0.176	0.234
Inflammatory Response	Hallmark	24	FDR < 0.05	0.384	0.001	0.012
TNFA/NF-kB	Hallmark	22	Tested in LMM	0.610	0.352	0.423
IL6/JAK/STAT3	Hallmark	0	No features at FDR < 0.10	—	—	—
IFN Gamma Response	Hallmark	0	No features at FDR < 0.10	—	—	—
P53 Pathway	Hallmark	23	Tested in LMM	0.533	0.101	0.223
MYC Targets V1	Hallmark	0	No features at FDR < 0.10	—	—	—
E2F Targets	Hallmark	0	No features at FDR < 0.10	—	—	—
G2M Checkpoint	Hallmark	0	No features at FDR < 0.10	—	—	—
mTORC1 Signaling	Hallmark	3	Tested in LMM	0.694	0.133	0.223
Glycolysis	Hallmark	21	Tested in LMM	0.804	0.112	0.223
Oxidative Phosphorylation	Hallmark	3	Tested in LMM	0.344	0.800	0.800
Complement	Hallmark	21	Tested in LMM	0.208	0.148	0.223
Neftel MES	Neftel	2	Tested in LMM	0.562	0.133	0.223
Neftel AC	Neftel	0	No features at FDR < 0.10	—	—	—
Neftel OPC	Neftel	0	No features at FDR < 0.10	—	—	—
Neftel NPC	Neftel	0	No features at FDR < 0.10	—	—	—
IvyGAP CT module	IvyGAP Zone	0	No features at FDR < 0.10	—	—	—
IvyGAP CTmvp module	IvyGAP Zone	0	No features at FDR < 0.10	—	—	—
IvyGAP CTpan module	IvyGAP Zone	5	Tested in LMM	0.902	0.773	0.800
IvyGAP IT module	IvyGAP Zone	0	No features at FDR < 0.10	—	—	—
IvyGAP LE module	IvyGAP Zone	0	No features at FDR < 0.10	—	—	—

Table S3. Full sensitivity analysis results for the Inflammatory Response pathway across all analysis variants. R^2_m = marginal R^2 (fixed effects only); LRT p = likelihood ratio test p-value comparing full model (radiomic features + subcompartment) to null model (subcompartment only). All variants use the same five radiomic features except where noted.

Variant	Description	R^2_m	LRT p	N patients	Significant (p < 0.05)
Primary	Default analysis	0.384	0.0010	28	Yes
S1a	ET = CT only (excluding MVP)	0.365	0.0026	28	Yes
S1b	Conservative zone mapping	0.421	0.0025	28	Yes
S2	Patients with >= 2 subcompartments only	0.412	0.0070	16	Yes
S3	Median aggregation (instead of mean)	0.366	0.0006	28	Yes
S7	Global standardization (instead of within-subcompartment)	0.317	0.0761	28	No
S8	Kenward-Roger denominator degrees of freedom	0.384	0.0062	28	Yes

Six of seven variants retained significance (LRT $p < 0.05$). The sole exception (S7, global standardization) approached significance ($p = 0.076$), consistent with attenuated signal when between-subcompartment variance is not removed prior to modeling.

Table S4. Gene set pairwise Jaccard similarity matrix (24 x 24). Values represent the Jaccard index (intersection/union of gene members) between gene sets. Higher values indicate greater overlap. Most pairs show minimal overlap (J < 0.10), confirming that the 24 gene sets capture largely distinct biological programs. Notable exceptions: IvyGAP IT and LE modules (J = 0.653); E2F Targets and G2M Checkpoint (J = 0.223); Glycolysis and Hypoxia (J = 0.194); IvyGAP CTpan and Hypoxia (J = 0.146); Inflammatory Response and TNFA/NF-kB (J = 0.146).

	**Hyp**	**Ang**	**EMT**	**IR**	**TNF**	**IL6**	**IFN**	**P53**	**MYC**	**E2F**	**G2M**	**mTO**	**Gly**	**OxP**	**Com**	**MES**	**AC**	**OPC**	**NPC**	**CT**	**mvp**	**pan**	**IT**	**LE**
**Hyp**	1.000	.013	.047	.028	.078	.014	.020	.036	.005	.005	.008	.084	.194	.005	.028	.037	.025	.000	.000	.011	.018	.146	.003	.000
**Ang**	.013	1.000	.054	.013	.017	.017	.000	.022	.000	.000	.000	.004	.013	.000	.009	.047	.000	.000	.000	.000	.017	.009	.000	.000
**EMT**	.047	.054	1.000	.034	.058	.029	.013	.018	.003	.003	.005	.005	.036	.000	.031	.139	.004	.004	.008	.004	.075	.047	.005	.003
**IR**	.028	.013	.034	1.000	.146	.087	.102	.031	.003	.005	.008	.013	.013	.000	.055	.037	.012	.000	.000	.000	.010	.026	.008	.008
**TNF**	.078	.017	.058	.146	1.000	.067	.072	.070	.003	.005	.013	.026	.013	.000	.042	.033	.008	.000	.000	.000	.003	.081	.000	.000
**IL6**	.014	.017	.029	.087	.067	1.000	.083	.025	.000	.000	.004	.004	.011	.000	.025	.022	.000	.000	.000	.000	.011	.025	.000	.000
**IFN**	.020	.000	.013	.102	.072	.083	1.000	.028	.005	.005	.008	.020	.008	.003	.061	.012	.004	.000	.000	.000	.008	.020	.000	.000
**P53**	.036	.022	.018	.031	.070	.025	.028	1.000	.008	.018	.003	.028	.013	.010	.010	.012	.004	.000	.000	.004	.005	.036	.010	.008
**MYC**	.005	.000	.003	.003	.003	.000	.005	.008	1.000	.096	.078	.050	.013	.028	.000	.000	.000	.000	.000	.000	.000	.005	.000	.000
**E2F**	.005	.000	.003	.005	.005	.000	.005	.018	.096	1.000	.223	.036	.018	.000	.000	.000	.000	.000	.004	.004	.000	.003	.000	.000
**G2M**	.008	.000	.005	.008	.013	.004	.008	.003	.078	.223	1.000	.023	.018	.000	.000	.008	.004	.000	.008	.015	.000	.003	.000	.000
**mTO**	.084	.004	.005	.013	.026	.004	.020	.028	.050	.036	.023	1.000	.072	.018	.013	.012	.004	.000	.004	.007	.005	.072	.003	.003
**Gly**	.194	.013	.036	.013	.013	.011	.008	.013	.013	.018	.018	.072	1.000	.020	.013	.020	.008	.000	.004	.004	.005	.070	.000	.000
**OxP**	.005	.000	.000	.000	.000	.000	.003	.010	.028	.000	.000	.018	.020	1.000	.008	.000	.000	.000	.000	.000	.000	.003	.003	.003
**Com**	.028	.009	.031	.055	.042	.025	.061	.010	.000	.000	.000	.013	.013	.008	1.000	.028	.012	.000	.000	.000	.015	.018	.008	.005
**MES**	.037	.047	.139	.037	.033	.022	.012	.012	.000	.000	.008	.012	.020	.000	.028	1.000	.000	.000	.000	.000	.037	.050	.000	.000
**AC**	.025	.000	.004	.012	.008	.000	.004	.004	.000	.000	.004	.004	.008	.000	.012	.000	1.000	.000	.000	.075	.000	.012	.012	.004
**OPC**	.000	.000	.004	.000	.000	.000	.000	.000	.000	.000	.000	.000	.000	.000	.000	.000	.000	1.000	.000	.084	.000	.000	.012	.004
**NPC**	.000	.000	.008	.000	.000	.000	.000	.000	.000	.004	.008	.004	.004	.000	.000	.000	.000	.000	1.000	.008	.000	.004	.045	.045
**CT**	.011	.000	.004	.000	.000	.000	.000	.004	.000	.004	.015	.007	.004	.000	.000	.000	.075	.084	.008	1.000	.000	.000	.007	.000
**mvp**	.018	.017	.075	.010	.003	.011	.008	.005	.000	.000	.000	.005	.005	.000	.015	.037	.000	.000	.000	.000	1.000	.000	.005	.000
**pan**	.146	.009	.047	.026	.081	.025	.020	.036	.005	.003	.003	.072	.070	.003	.018	.050	.012	.000	.004	.000	.000	1.000	.000	.000
**IT**	.003	.000	.005	.008	.000	.000	.000	.010	.000	.000	.000	.003	.000	.003	.008	.000	.012	.012	.045	.007	.005	.000	1.000	.653
**LE**	.000	.000	.003	.008	.000	.000	.000	.008	.000	.000	.000	.003	.000	.003	.005	.000	.004	.004	.045	.000	.000	.000	.653	1.000

Column abbreviations: Hyp = Hypoxia, Ang = Angiogenesis, EMT = Epithelial-Mesenchymal Transition, IR = Inflammatory Response, TNF = TNFA/NF-kB, IL6 = IL6/JAK/STAT3, IFN = IFN Gamma Response, P53 = P53 Pathway, MYC = MYC Targets V1, E2F = E2F Targets, G2M = G2M Checkpoint, mTO = mTORC1 Signaling, Gly = Glycolysis, OxP = Oxidative Phosphorylation, Com = Complement, MES = Neftel MES, AC = Neftel AC, OPC = Neftel OPC, NPC = Neftel NPC, CT = IvyGAP CT module, mvp = IvyGAP CTmvp module, pan = IvyGAP CTpan module, IT = IvyGAP IT module, LE = IvyGAP LE module.

Table S5. Legacy pre-screened Elastic Net results compared with nested CV results. The legacy analysis pre-selected features on the full dataset before LOPO-CV, introducing data leakage. Only pathways with $R^2_{cv} > 0$ in either analysis are shown.

Pathway	Legacy pre-screened R^2	Legacy S5 R^2	Nested R^2_{cv}
Angiogenesis	—	—	0.209
Inflammatory Response	-0.104	0.180	0.185
IvyGAP CTpan module	—	—	0.133

Table S6. Feature lookup table mapping radiomic feature indices to full IBSI names, MRI sequence, and feature type. All 89 unique radiomic features that entered any model across all 24 pathways are listed.

Feature ID	IBSI Feature Name	Sequence	Feature Type
rad_71	T1 kurtosis-CoLIAGe correlation ws=5	T1	Higher-order (CoLIAGe)
rad_126	T1 Histogram Bins-16 Bin-0 Probability	T1	First-order (Histogram)
rad_163	T1 Histogram Bins-16 Kurtosis	T1	First-order (Histogram)
rad_166	T1 Histogram Bins-16 MeanAbsoluteDeviation	T1	First-order (Histogram)
rad_227	T1 GLSZM Bins-16 Radius-1 GreyLevelNonUniformityNormalized	T1	Texture (GLSZM)
rad_240	T1 GLSZM Bins-16 Radius-1 ZoneSizeNonUniformity	T1	Texture (GLSZM)
rad_249	T1 Histogram Bins-64 Bin-0 Frequency	T1	First-order (Histogram)
rad_418	T1 GLRLM Bins-64 Radius-1 LongRunLowGreyLevelEmphasis	T1	Texture (GLRLM)
rad_439	T1 GLSZM Bins-64 Radius-1 ZoneSizeNonUniformity	T1	Texture (GLSZM)
rad_565	T1 Histogram Bins-128 Bin-3 Probability	T1	First-order (Histogram)
rad_601	T1 Histogram Bins-128 Bin-56 Probability	T1	First-order (Histogram)
rad_611	T1 Histogram Bins-128 Bin-60 Probability	T1	First-order (Histogram)
rad_733	T1 GLCM Bins-128 Radius-1 Entropy	T1	Texture (GLCM)
rad_1000	T2 skewness-CoLIAGe correlation ws=3	T2	Higher-order (CoLIAGe)
rad_1049	T2 median-CoLIAGe correlation ws=5	T2	Higher-order (CoLIAGe)
rad_1051	T2 kurtosis-CoLIAGe correlation ws=5	T2	Higher-order (CoLIAGe)
rad_1052	T2 skewness-CoLIAGe correlation ws=5	T2	Higher-order (CoLIAGe)
rad_1062	T2 var-CoLIAGe sum-av ws=5	T2	Higher-order (CoLIAGe)
rad_1064	T2 skewness-CoLIAGe sum-av ws=5	T2	Higher-order (CoLIAGe)
rad_1067	T2 kurtosis-CoLIAGe sum-var ws=5	T2	Higher-order (CoLIAGe)
rad_1074	T2 var-CoLIAGe diff-av ws=5	T2	Higher-order (CoLIAGe)
rad_1076	T2 skewness-CoLIAGe diff-av ws=5	T2	Higher-order (CoLIAGe)
rad_1079	T2 kurtosis-CoLIAGe diff-var ws=5	T2	Higher-order (CoLIAGe)
rad_1080	T2 skewness-CoLIAGe diff-var ws=5	T2	Higher-order (CoLIAGe)
rad_1148	T2 Histogram Bins-16 MedianAbsoluteDeviation	T2	First-order (Histogram)
rad_1220	T2 GLSZM Bins-16 Radius-1 ZoneSizeNonUniformity	T2	Texture (GLSZM)

Feature ID	IBSI Feature Name	Sequence	Feature Type
rad_1226	T2 NGTDM Contrast	T2	Texture (NGTDM)
rad_1251	T2 Histogram Bins-64 Bin-1 Frequency	T2	First-order (Histogram)
rad_1252	T2 Histogram Bins-64 Bin-1 Probability	T2	First-order (Histogram)
rad_1296	T2 Histogram Bins-64 Bin-3 Probability	T2	First-order (Histogram)
rad_1363	T2 Histogram Bins-64 Kurtosis	T2	First-order (Histogram)
rad_1422	T2 Histogram Bins-128 Bin-0 Frequency	T2	First-order (Histogram)
rad_1501	T2 Histogram Bins-128 Bin-1 Probability	T2	First-order (Histogram)
rad_1523	T2 Histogram Bins-128 Bin-2 Probability	T2	First-order (Histogram)
rad_1567	T2 Histogram Bins-128 Bin-4 Probability	T2	First-order (Histogram)
rad_1707	T2 GLCM Bins-128 Radius-1 AutoCorrelation	T2	Texture (GLCM)
rad_1732	T2 GLSZM Bins-128 Radius-1 LargeZoneLowGreyLevelEmphasis	T2	Texture (GLSZM)
rad_1763	T2 GLSZM Bins-16 Radius-2 GreyLevelNonUniformityNormalized	T2	Texture (GLSZM)
rad_1872	T2 GLSZM Bins-16 Radius-3 GreyLevelNonUniformityNormalized	T2	Texture (GLSZM)
rad_1920	T2 GLSZM Bins-64 Radius-3 ZoneSizeEntropy	T2	Texture (GLSZM)
rad_1930	T2 GLCM Bins-128 Radius-3 Energy	T2	Texture (GLCM)
rad_1931	T2 GLCM Bins-128 Radius-3 Entropy	T2	Texture (GLCM)
rad_1945	T2 GLSZM Bins-128 Radius-3 GreyLevelNonUniformityNormalized	T2	Texture (GLSZM)
rad_1950	T2 GLSZM Bins-128 Radius-3 LargeZoneLowGreyLevelEmphasis	T2	Texture (GLSZM)
rad_1956	T2 GLSZM Bins-128 Radius-3 ZoneSizeEntropy	T2	Texture (GLSZM)
rad_1977	T1ce median-CoLIAGe correlation ws=3	T1ce	Higher-order (CoLIAGe)
rad_1978	T1ce var-CoLIAGe correlation ws=3	T1ce	Higher-order (CoLIAGe)
rad_1979	T1ce kurtosis-CoLIAGe correlation ws=3	T1ce	Higher-order (CoLIAGe)
rad_2002	T1ce var-CoLIAGe diff-av ws=3	T1ce	Higher-order (CoLIAGe)
rad_2003	T1ce kurtosis-CoLIAGe diff-av ws=3	T1ce	Higher-order (CoLIAGe)
rad_2007	T1ce kurtosis-CoLIAGe diff-var ws=3	T1ce	Higher-order (CoLIAGe)
rad_2023	T1ce kurtosis-CoLIAGe inertia ws=5	T1ce	Higher-order (CoLIAGe)
rad_2028	T1ce skewness-CoLIAGe idm ws=5	T1ce	Higher-order (CoLIAGe)
rad_2030	T1ce var-CoLIAGe correlation ws=5	T1ce	Higher-order (CoLIAGe)

Feature ID	IBSI Feature Name	Sequence	Feature Type
rad_2031	T1ce kurtosis-CoLIAGe correlation ws=5	T1ce	Higher-order (CoLIAGe)
rad_2032	T1ce skewness-CoLIAGe correlation ws=5	T1ce	Higher-order (CoLIAGe)
rad_2044	T1ce skewness-CoLIAGe sum-av ws=5	T1ce	Higher-order (CoLIAGe)
rad_2048	T1ce skewness-CoLIAGe sum-var ws=5	T1ce	Higher-order (CoLIAGe)
rad_2055	T1ce kurtosis-CoLIAGe diff-av ws=5	T1ce	Higher-order (CoLIAGe)
rad_2056	T1ce skewness-CoLIAGe diff-av ws=5	T1ce	Higher-order (CoLIAGe)
rad_2073	T1ce Intensity MedianAbsoluteDeviation	T1ce	Other
rad_2074	T1ce Intensity Minimum	T1ce	Other
rad_2077	T1ce Intensity QuartileCoefficientOfVariation	T1ce	Other
rad_2134	T1ce Histogram Bins-16 QuartileCoefficientOfVariation	T1ce	First-order (Histogram)
rad_2198	T1ce GLSZM Bins-16 Radius-1 ZoneSizeEntropy	T1ce	Texture (GLSZM)
rad_2209	T1ce Histogram Bins-64 Bin-0 Frequency	T1ce	First-order (Histogram)
rad_2397	T1ce GLSZM Bins-64 Radius-1 ZoneSizeEntropy	T1ce	Texture (GLSZM)
rad_2481	T1ce Histogram Bins-128 Bin-1 Probability	T1ce	First-order (Histogram)
rad_2495	T1ce Histogram Bins-128 Bin-26 Probability	T1ce	First-order (Histogram)
rad_2511	T1ce Histogram Bins-128 Bin-33 Probability	T1ce	First-order (Histogram)
rad_2635	T1ce Histogram Bins-128 Bin-8 Probability	T1ce	First-order (Histogram)
rad_2662	T1ce Histogram Bins-128 FifthPercentileMean	T1ce	First-order (Histogram)
rad_2711	T1ce GLSZM Bins-128 Radius-1 LargeZoneHighGreyLevelEmphasis	T1ce	Texture (GLSZM)
rad_2718	T1ce GLSZM Bins-128 Radius-1 ZoneSizeEntropy	T1ce	Texture (GLSZM)
rad_2794	T1ce GLSZM Bins-64 Radius-2 ZoneSizeNoneUniformityNormalized	T1ce	Texture (GLSZM)
rad_2910	T1ce GLCM Bins-128 Radius-3 Energy	T1ce	Texture (GLCM)
rad_3064	FLAIR Intensity Variance	FLAIR	Other
rad_3066	FLAIR Histogram Bins-16 Bin-0 Probability	FLAIR	First-order (Histogram)
rad_3131	FLAIR Morphologic EllipseDiameter Axis-2	FLAIR	Other
rad_3180	FLAIR GLSZM Bins-16 Radius-1 ZoneSizeNonUniformity	FLAIR	Texture (GLSZM)
rad_3234	FLAIR Histogram Bins-64 Bin-2 Probability	FLAIR	First-order (Histogram)
rad_3375	FLAIR GLSZM Bins-64 Radius-1 SmallZoneLowGreyLevelEmphasis	FLAIR	Texture (GLSZM)
rad_3549	FLAIR Histogram Bins-128 Bin-5 Probability	FLAIR	First-order (Histogram)

Feature ID	IBSI Feature Name	Sequence	Feature Type
rad_3571	FLAIR Histogram Bins-128 Bin-6 Probability	FLAIR	First-order (Histogram)
rad_3647	FLAIR Histogram Bins-128 MeanAbsoluteDeviation	FLAIR	First-order (Histogram)
rad_3813	FLAIR GLCM Bins-16 Radius-3 ClusterProminence	FLAIR	Texture (GLCM)
rad_3848	FLAIR LBP Radius-3 LBP	FLAIR	Other
rad_3880	FLAIR GLSZM Bins-64 Radius-3 ZoneSizeEntropy	FLAIR	Texture (GLSZM)
rad_3909	FLAIR GLSZM Bins-128 Radius-3 LargeZoneHighGreyLevelEmphasis	FLAIR	Texture (GLSZM)

Table S7. Clinical covariate adjustment results for the Inflammatory Response pathway (sensitivity analysis S9; Section 2.9). Progressive covariate adjustment demonstrating that radiomic features remain significant after accounting for age, MGMT methylation status, and molecular subtype. LRT tests the contribution of the five radiomic features above the covariates included in each model.

Model	Covariates	R^2_m	R^2_c	LRT chi^2	LRT p	N obs	N patients	Converged
A: Primary	Subcompartment only	0.384	0.687	20.53	0.0010	50	28	Yes
B: + age	Subcompartment + age	0.378	0.702	20.03	0.0012	50	28	Yes
C: + age + MGMT	Subcompartment + age + MGMT	0.369	0.719	19.26	0.0017	50	28	Yes
D: + age + MGMT + subtype	Subcompartment + age + MGMT + molecular subtype	0.479	0.785	17.51	0.0036	50	28	Yes

Radiomic features remain significant (LRT $p < 0.005$) in all four models. R^2_m = marginal R^2 (fixed effects only); R^2_c = conditional R^2 (fixed + random effects); LRT = likelihood ratio test comparing full model (with radiomic features) to reduced model (covariates only).

Individual radiomic feature coefficients across adjustment models:

Model A: Primary (no clinical covariates)

Feature	IBSI Name	Beta	SE	95% CI	p (Holm)
rad_1732	T2 GLSZM Bins-128 Radius-1 LargeZoneLowGreyLevelEmphasis	-0.471	0.141	[-0.758, -0.184]	0.010 *
rad_1080	T2 skewness-CoLIAGe diff-var ws=5	0.308	0.149	[0.007, 0.610]	0.181
rad_1251	T2 Histogram Bins-64 Bin-1 Frequency	-0.180	0.110	[-0.402, 0.042]	0.326
rad_1052	T2 skewness-CoLIAGe correlation ws=5	0.130	0.163	[-0.199, 0.459]	0.858
rad_1950	T2 GLSZM Bins-128 Radius-3 LargeZoneLowGreyLevelEmphasis	0.003	0.159	[-0.321, 0.328]	0.984

Model D: Fully adjusted (+ age + MGMT + molecular subtype)

Feature	IBSI Name	Beta	SE	95% CI	p (Holm)
rad_1732	T2 GLSZM Bins-128 Radius-1 LargeZoneLowGreyLevelEmphasis	-0.471	0.138	[-0.753, -0.188]	0.010 *
rad_1080	T2 skewness-CoLIAGe diff-var ws=5	0.295	0.150	[-0.011, 0.601]	0.232
rad_1052	T2 skewness-CoLIAGe correlation ws=5	0.239	0.170	[-0.108, 0.585]	0.510
rad_1251	T2 Histogram Bins-64 Bin-1 Frequency	-0.119	0.108	[-0.339, 0.101]	0.558
rad_1950	T2 GLSZM Bins-128 Radius-3 LargeZoneLowGreyLevelEmphasis	0.068	0.154	[-0.248, 0.383]	0.665

* Holm-adjusted $p < 0.05$. rad_1732 (T2 GLSZM LargeZoneLowGreyLevelEmphasis) is the only individually significant feature, remaining significant across all four adjustment models.

Table S8. CLEAR (CheckList for EvaluAtion of Radiomics research) compliance table. Self-assessment following CLEAR v1.0 [32].

Item	Domain	Description	Compliance	Section	Notes
1	Title	Indicate use of radiomics in title	Yes	Title	"Radiomic Features" in title
2	Abstract	Structured summary with methods, results, uncertainty	Yes	Abstract	R^2_{cv} with 95% CIs and permutation p-values
3	Keywords	Keywords indicating radiomics study	Yes	Keywords	"radiomics" and related terms
4	Introduction	Scientific/clinical problem with literature review	Yes	Section 1	GBM heterogeneity, prior work reviewed
5	Introduction	Rationale for radiomic approach	Yes	Section 1	Zone-to-subcompartment mapping rationale
6	Introduction	Study objectives	Yes	Section 1	Hypothesis-generating analysis stated
7	Study Design	Indicate CLEAR checklist use	Yes	Section 2.11	CLEAR compliance declared
8	Study Design	Ethical approval	Yes	IRB Statement	Public datasets, no IRB required
9	Study Design	Sample size calculation	Yes	Section 2.10	Riley criterion: N_{min} = 240, acknowledged underpowered
10	Study Design	Study nature	Yes	Section 2.1	Retrospective analysis of public datasets
11	Study Design	Inclusion/exclusion criteria	Partial	Section 2.1	Patient matching described
12	Study Design	Flowchart of methodology	Yes	Figures 1-2	Data flow and feature reduction shown
13	Data	Data source with links	Yes	Section 2.1	IvyGAP and TCIA cited
14	Data	Prior dataset usage declared	Yes	Section 1	Le et al., Park et al. discussed
15	Data	Data split with leakage prevention	Yes	Sections 2.6-2.7	Nested LOPO-CV with internal feature selection
16	Data	Imaging protocol and scanner	Partial	Section 2.1	Deferred to Pati et al.
17	Data	Non-radiomic predictor variables	Yes	Section 2.8	Age, MGMT, molecular subtype (S9)
18	Data	Reference standard/outcome measure	Yes	Section 2.3	ssGSEA pathway enrichment scores
19	Segmentation	Segmentation software and method	Partial	Section 2.1	BraTS-style from Pati et al.
20	Segmentation	Number of readers and experience	Partial	Section 2.1	Multi-expert per Pati et al.
21	Pre-processing	Pre-processing software and parameters	N/A	Section 2.1	Pre-extracted features
22	Pre-processing	Resampling technique	N/A	—	Details in Pati et al.
23	Pre-processing	Discretization method	N/A	—	Multiple bin configurations
24	Pre-processing	Image types and filter parameters	N/A	—	4 MRI sequences
25	Feature Extraction	Feature extraction software, IBSI	Yes	Section 2.1	IBSI-compliant, CaPTk

Item	Domain	Description	Compliance	Section	Notes
26	Feature Extraction	Feature classes using IBSI terminology	Yes	Table 3, S6	GLCM, GLSZM, Histogram, CoLIAGe
27	Feature Extraction	Total features per instance	Yes	Section 2.1	3920 per subcompartment
28	Feature Extraction	Default parameters stated	N/A	—	Pre-extracted features
29	Data Preparation	Missing data handling	Partial	Section 2.4	Near-zero-variance removal
30	Data Preparation	Class balance	N/A	—	Regression task
31	Data Preparation	Segmentation reliability	Partial	Section 2.1	Reproducibility in Pati et al.
32	Data Preparation	Feature normalization	Yes	Sections 2.4-2.5	Within-subcompartment z-scoring
33	Data Preparation	Dimension reduction	Yes	Section 2.4	Three-stage pipeline
34	Modeling	Software and algorithm details	Yes	Sections 2.5-2.6	lme4, glmnet with versions
35	Modeling	Training process and hyperparameters	Yes	Section 2.6	Alpha grid, inner 5-fold CV, lambda.1se
36	Modeling	Confounder detection	Yes	Section 2.8	Progressive covariate adjustment (S9)
37	Modeling	Final model selection	Yes	Section 2.6	Conservative lambda.1se rule
38	Evaluation	Internal or external testing	Yes	Sections 2.6, 2.12	Internal LOPO-CV only
39	Evaluation	Performance metrics	Yes	Section 2.6	R^2_{cv}, MAE, Spearman rho
40	Evaluation	Uncertainty quantification	Yes	Section 2.6	Bootstrap CIs, permutation p-values
41	Evaluation	Statistical software and comparison	Yes	Sections 2.5, 2.7	LRT, FDR, Holm corrections
42	Evaluation	Comparison with non-radiomic approaches	Yes	Section 2.8	Clinical covariate-only models
43	Evaluation	Interpretability/explainability	Partial	Section 3.3	Feature stability selection
44	Results	Baseline characteristics	Partial	Section 3.1	Sample counts reported
45	Results	Flowchart with inclusion/exclusion	Yes	Section 3.1	41 -> 31 -> 28 patients
46	Results	Feature statistics and selection	Yes	Tables 3, S6, S12	Stability, IBSI names, coefficients
47	Results	Performance metrics for train/val/test	Yes	Tables 2, 3	Nested CV R^2_{cv} with CIs
48	Results	Comparison with non-radiomic approaches	Yes	S7	Radiomic contribution above covariates
49	Discussion	Summary and study categorization	Yes	Discussion	Exploratory/hypothesis-generating
50	Discussion	Comparison with previous works	Yes	Discussion	Park, Le, Hu, Zhang compared
51	Discussion	Practical implications and future	Yes	Discussion	Speculative nature acknowledged
52	Discussion	Strengths and limitations	Yes	Limitations	10 limitations enumerated

Item	Domain	Description	Compliance	Section	Notes
53	Open Science	Raw/processed image data shared	N/A	Data Availability	Public datasets
54	Open Science	Radiomic feature data shared	Yes	Data Availability	Pre-extracted from TCIA
55	Open Science	Pre-processing/extraction scripts	N/A	—	Pre-extracted features
56	Open Science	Modeling scripts shared	Yes	Section 2.13	Code on GitHub
57	Open Science	Final model files shared	Partial	Section 2.13	Code shared, not serialized models
58	Open Science	Ready-to-use tool	No	—	Exploratory study

CLEAR summary: 40 Yes, 8 Partial, 8 N/A, 1 No (out of 57 items; the single “No” reflects the exploratory nature of the study).

Table S9. Top radiomic features for the Angiogenesis pathway, identified by stability selection across 28 LOPO folds. Features selected in >50% of folds are considered stable.

Feature ID	IBSI Feature Name	Folds selected	Stability (%)
rad_1763	T2 GLSZM Bins-16 Radius-2 GreyLevelNonUniformityNormalized	28	100.0
rad_1872	T2 GLSZM Bins-16 Radius-3 GreyLevelNonUniformityNormalized	28	100.0
rad_1920	T2 GLSZM Bins-64 Radius-3 ZoneSizeEntropy	28	100.0
rad_1363	T2 Histogram Bins-64 Kurtosis	27	96.4
rad_1956	T2 GLSZM Bins-128 Radius-3 ZoneSizeEntropy	27	96.4
rad_163	T1 Histogram Bins-16 Kurtosis	1	3.6
rad_2077	T1ce Intensity QuartileCoefficientOfVariation	1	3.6

Table S10. BH-FDR-corrected nested CV permutation p-values for all 24 pathways. Pathways with R^2_{cv} <= 0 were assigned p = 1.0 before FDR correction (conservative approach).

**Pathway**	R^2_{cv}	**Permutation p (raw)**	**Permutation p (FDR)**
Angiogenesis	0.209	0.006	0.096
Inflammatory Response	0.185	0.008	0.096
IvyGAP CTpan module	0.133	0.013	0.104
Hypoxia	-0.135	1.000	1.000
EMT	-0.034	1.000	1.000
TNFA/NF-kB	-0.113	1.000	1.000
IL6/JAK/STAT3	-0.199	1.000	1.000
IFN Gamma Response	-0.210	1.000	1.000
P53 Pathway	-0.075	1.000	1.000
MYC Targets V1	-0.075	1.000	1.000
E2F Targets	-0.075	1.000	1.000
G2M Checkpoint	-0.075	1.000	1.000
mTORC1 Signaling	-0.075	1.000	1.000
Glycolysis	-0.109	1.000	1.000
Oxidative Phosphorylation	-0.075	1.000	1.000
Complement	-0.004	1.000	1.000
Neftel MES	-0.146	1.000	1.000
Neftel AC	-0.075	1.000	1.000
Neftel OPC	-0.086	1.000	1.000
Neftel NPC	-0.075	1.000	1.000
IvyGAP CT module	-0.075	1.000	1.000
IvyGAP CTmvp module	-0.274	1.000	1.000
IvyGAP IT module	-0.072	1.000	1.000
IvyGAP LE module	-0.114	1.000	1.000

Table S11. Hyperparameter distributions (alpha, lambda) across LOPO folds for the three pathways with positive predictive signal. Alpha was selected from a grid of 0.1 to 1.0 (step 0.1); lambda was selected using the 1-SE rule (lambda.1se) from inner 5-fold CV.

Pathway	Folds with features	Alpha (min)	Alpha (max)	Alpha (median)	Lambda (min)	Lambda (max)	Lambda (median)
Angiogenesis	28	0.1	1.0	0.5	0.264	2.506	0.656
Inflammatory Response	28	0.1	1.0	0.4	0.270	1.637	0.663
IvyGAP CTpan module	28	0.1	1.0	0.6	0.175	1.698	0.455

Table S12. Top radiomic features for the Inflammatory Response pathway, identified by stability selection across 28 LOPO folds. Full IBSI feature names, fold selection counts, and stability percentages are provided. Features selected in >50% of folds are considered stable.

Feature ID	IBSI Feature Name	Folds selected	Stability (%)
rad_1707	T2 GLCM Bins-128 Radius-1 AutoCorrelation	28	100.0
rad_1950	T2 GLSZM Bins-128 Radius-3 LargeZoneLowGreyLevelEmphasis	28	100.0
rad_1930	T2 GLCM Bins-128 Radius-3 Energy	27	96.4
rad_1945	T2 GLSZM Bins-128 Radius-3 GreyLevelNonUniformityNormalized	20	71.4
rad_1363	T2 Histogram Bins-64 Kurtosis	16	57.1
rad_3909	FLAIR GLSZM Bins-128 Radius-3 LargeZoneHighGreyLevelEmphasis	4	14.3
rad_1000	T2 skewness-CoLIAGe correlation ws=3	2	7.1
rad_2134	T1ce Histogram Bins-16 QuartileCoefficientOfVariation	1	3.6
rad_2635	T1ce Histogram Bins-128 Bin-8 Probability	1	3.6

Table S13. Coefficient-level results for the Inflammatory Response linear mixed-effects model. Standardized beta coefficients, standard errors, 95% confidence intervals, Type II Satterthwaite ANOVA F-values, and Holm-adjusted p-values for all five radiomic features and the subcompartment fixed effect. Model: $R^2_m = 0.384$, $R^2_c = 0.687$, LRT $chi^2 = 20.53$, df = 5, p = 0.001.

Term	Beta	SE	95% CI	F	df	p	p (Holm)
rad_1732 (T2 GLSZM Bins-128 R1 LargeZoneLowGreyLevelEmphasis)	-0.471	0.141	[-0.758, -0.184]	11.16	33.0	0.0021	0.010 *
rad_1080 (T2 skewness-CoLIAGe diff-var ws=5)	0.308	0.149	[0.007, 0.610]	4.26	40.8	0.0454	0.181
rad_1251 (T2 Histogram Bins-64 Bin-1 Frequency)	-0.180	0.110	[-0.402, 0.042]	2.70	37.9	0.1088	0.326
rad_1052 (T2 skewness-CoLIAGe correlation ws=5)	0.130	0.163	[-0.199, 0.459]	0.64	40.3	0.4291	0.858
rad_1950 (T2 GLSZM Bins-128 R3 LargeZoneLowGreyLevelEmphasis)	0.003	0.159	[-0.321, 0.328]	0.00	32.9	0.9842	0.984
Subcompartment (NET vs ET)	1.945	0.093	[1.755, 2.135]	9.48[a]	17.5	0.0016	—
Subcompartment (ED vs ET)	-0.577	0.274	[-1.152, -0.001]	—	17.9	0.0496	—

* Holm-adjusted p < 0.05 among the five radiomic features.

[a] Joint F-test for the subcompartment factor (2 df). Individual subcompartment contrasts are reported as t-tests. The subcompartment effect accounts for R^2_m (null) = 0.170; the radiomic increment is $\Delta R^2_m = 0.214$. All features are T2-derived. Coefficients are standardized (z-scored predictors).
